# GBScleanR: robust genotyping error correction using a hidden Markov model with error pattern recognition

**DOI:** 10.1093/genetics/iyad055

**Published:** 2023-03-29

**Authors:** Tomoyuki Furuta, Toshio Yamamoto, Motoyuki Ashikari

**Affiliations:** Institute of Plant Science and Resources, Okayama University, Chu-oh 2-20-1, Kurashiki, Okayama 710-0046, Japan; Institute of Plant Science and Resources, Okayama University, Chu-oh 2-20-1, Kurashiki, Okayama 710-0046, Japan; Bioscience and Biotechnology Center, Nagoya University, Furo-cho, Chikusa, Nagoya, Aichi 464-8601, Japan

**Keywords:** reduced-representation sequencing, error correction, imputation, hidden Markov model

## Abstract

Reduced-representation sequencing (RRS) provides cost-effective and time-saving genotyping platforms. Despite the outstanding advantage of RRS in throughput, the obtained genotype data usually contain a large number of errors. Several error correction methods employing the hidden Markov model (HMM) have been developed to overcome these issues. These methods assume that markers have a uniform error rate with no bias in the allele read ratio. However, bias does occur because of uneven amplification of genomic fragments and read mismapping. In this paper, we introduce an error correction tool, GBScleanR, which enables robust and precise error correction for noisy RRS-based genotype data by incorporating marker-specific error rates into the HMM. The results indicate that GBScleanR improves the accuracy by more than 25 percentage points at maximum compared to the existing tools in simulation data sets and achieves the most reliable genotype estimation in real data even with error-prone markers.

## Introduction

Reduced-representation sequencing (RRS) is a sequencing technique using next-generation sequencing (NGS) with reducing sequence targets by taking reads only from a limited portion of a genome (Scheben *et al.* 2017). To meet the demands for cost-effective genotyping systems with dense markers, many different RRS-based genotyping methods have been introduced, including restriction site-associated DNA sequencing (RAD-seq) and genotyping-by-sequencing (GBS) (Baird *et al.* 2008; Elshire *et al.* 2011). The reduction in the number of sequence targets allows us for highly multiplexed sequencing to obtain genotype data of a large population such as a hybrid population for genetic mapping and breeding. However, a larger number of samples per NGS run result in genotype data with a lower read coverage per sample (Poland and Rife 2012). Since sequence reads that can be retrieved via NGS are highly stochastic, the limited number of reads can result in the undercalling of heterozygotes, in which recovering only 1 allele at a heterozygous site leads to its incorrect identification as a homozygote. The lack of reads also generates a substantial number of missing genotype calls.

Several error correction tools have been developed to overcome these disadvantages. The method to fill up missing calls and correct the undercalling of heterozygotes was first published in 2009, which determines genotypes based on the allele read ratio within a sliding window (Huang *et al.* 2009). Genotype-Corrector is also an example of the tool using a sliding window method for error correction (Miao *et al.* 2018). Recent publications have introduced statistically sophisticated methods using hidden Markov models (HMMs), such as FSFHap, TIGER, LB-Impute, and magicImpute (Swarts *et al.* 2014; Rowan *et al.* 2015; Fragoso *et al.* 2016; Zheng *et al.* 2018). The existing methods, however, assume constant error rates for all markers. For example, those assume a 50:50 probability that a read could be obtained for 1 of 2 possible alleles at a heterozygous site. Although this assumption is true in an ideal situation, in practice, genotype data contain a significant number of error-prone markers that show skewed probabilities in allele read acquisition as a result of actual biological unevenness (Davey *et al.* 2013; Wijnker *et al.* 2013; DaCosta and Sorenson 2014). Variations in genome sequences change the fragmentation patterns of the genomes. Even if 2 independent reads are mapped at the same locus, the sequences of the genomic fragments from which each of the 2 reads originated can vary in terms of GC content and length and sometimes include large insertions or deletions in the unsequenced region of the fragments. Since the GC contents and the lengths of the restriction fragments are known to affect the amplification efficiency, the probability of observing a read for either allele may differ. These biases are likely to be more prominent in a polymorphism-rich population, for example, that derived from a cross between distant relatives. Hence, mismatches occur between the real data, which shows marker-specific error rates, and the models that assume a uniform error rate, resulting in biased genotype estimation and poor error correction accuracy.

Here, we introduce the R package “GBScleanR,” which implements an HMM-based error correction algorithm. Our algorithm estimates an allele read bias and mismapping rate for each marker and incorporates those into the HMM as parameters to capture the skewed probabilities in allele read acquisitions. This paper demonstrates a comparison of GBScleanR and 2 well-established error correction tools: LB-Impute and magicImpute (Fragoso *et al.* 2016; Zheng *et al.* 2018). While LB-Impute accepts only biparental populations derived from inbred founders, magicImpute can work on biparental and multiparental populations derived from both inbred and outbred founders. The magicImpute algorithm is also able to estimate founder genotypes simultaneously with the offspring genotypes. Similar to magicImpute, GBScleanR supports simultaneous estimation of founder and offspring genotypes in a biparental and multiparental population. We first show simulation studies to present the accuracy and robustness of GBScleanR using simulation data sets that have severe allele read biases at error-prone markers. The simulation assumes 3 scenarios: a biparental *F*_2_ population (homoP2_F2), an outbred *F*_1_ population (hetP2_F1), and 8-way recombinant inbred lines (homoP8_RIL). We then demonstrate the reliability and robustness of GBScleanR using real data derived from a cross between distant relatives of rice, which potentially contains many error-prone markers.

## Methods

### Modeling for error correction

Our modeling basically follows the model implemented in magicImpute that has been introduced in Zheng *et al*. (2018). The error correction algorithm of GBScleanR employs the HMM and treats the observed allele read counts for each SNP marker along a chromosome as outputs from a sequence of latent true genotypes. Our model supposes that a population of $N^{o}\geq 1$ offspring is originally derived from crosses between $N^{f}\geq 2\;$ founder individuals. The founders can be inbred lines with homozygotes at all markers or outbred lines in which markers would be heterozygous. Only 1 chromosome is considered for modeling due to the independence of chromosomes. Let $Y^{o}=\;\{y_{mi}^{o}{\}}_{m=1\ldots M,i=1\ldots N^{o}}$ denote the observed allele read counts at marker *m* in offspring *i*. The element $y_{mi}^{o}$ consists of 2 values, $y^{\mathrm{ref}}$ and $y^{\mathrm{alt}}$, which represent the reference read count and the alternative read count, respectively. Similarly, the observed allele read counts at marker *m* in founder *j* are represented by $Y^{f}=\;\{y_{mj}^{f}{\}}_{m=1\ldots M,j=1\ldots N^{f}}$. The matrices for hidden true offspring genotypes and hidden true founder genotypes are represented by $X^{o}=\;\{x_{mi}^{o}{\}}_{m=1\ldots M,i=1\ldots N^{o}}$ and $X^{f}=\;\{x_{mj}^{f}{\}}_{m=1\ldots M,j=1\ldots N^{f}}$, respectively. The element $x_{mi}^{o}$ takes a value of 0, 1, or 2 to indicate the reference homozygote, heterozygote, and alternative homozygote genotype without phasing information. Unlike the offspring genotype matrix, the founder genotype $x_{mj}^{f}$ stores the phased genotype as $x_{mj}^{f}\;=\;(x^{1},x^{2})$, where $x^{1}\;$ and $x^{2}$ indicate alleles at marker *m* on one of the diploid chromosomes and another in founder *j*, respectively. The reference allele is represented by 0, while 1 denotes the alternative. Considering the linkage between the markers and the independence of the founder genotypes, we can assume that the sequence of the descendent genotypes from founders does not follow a Markov process, while the sequence of the descendent haplotypes $h_{i}^{o}$ does. To enable our estimation problem to be solved in the HMM framework, $H^{o}=\;\{h_{mi}^{o}{\}}_{m=1\ldots M,i=1\ldots N^{o}}$ is introduced to represent the matrix of the phased descendent haplotypes. The element $h_{mi}^{o}$ denotes a pair of descendent haplotypes $\left(h^{1},h^{2}\right)$ at marker *m* in offspring *i*. Therefore, $h^{1}$ and $h^{2}$ each take one of the natural numbers that are $\leq N^{f}$ if all founders are inbred or $\leq 2N^{f}$ if outbred to indicate the origins of the descendent haplotypes.

The algorithm estimates $H^{o}$ and $X^{f}$ based on $Y^{o}$ and $Y^{f}$ by maximizing the joint probability $P(H^{o},X^{f},Y^{o},Y^{f})$. We assume the independence of the offspring and the founders and the independence of each founder genotype at each marker. Therefore, our algorithm tries to maximize the following joint probability:


$$P(H^{o},X^{f},Y^{o},Y^{f})\;=\overset{N^{o}}{\underset{i=1}{\prod }}\;\left\{\overset{M}{\underset{m=1}{\prod }}\;P(y_{mi}^{o}|h_{mi}^{o},x_{m}^{f})\overset{M}{\underset{m=2}{\prod }}\;P(h_{mi}^{o}|h_{m-1,i}^{o},x_{m-1}^{f})P(h_{1i}^{o}|x_{1}^{f})\right\}$$

$$\times \overset{M}{\underset{m=1}{\prod }}\;P(y_{m}^{f}|x_{m}^{f})P(x_{m}^{f}),$$

where $P(y_{mi}^{o}|h_{mi}^{o},x_{m}^{f})$, $P(h_{mi}^{o}|h_{m-1,i}^{o},x_{m-1}^{f})$, and $P(h_{1i}^{o}|x_{1}^{f})$ correspond to the emission probability, the transition probability, and the initial probability of the HMM, respectively. $P(y_{m}^{f}|x_{m}^{f})$ is the probability of observing read counts for the founders at marker *m* when the combination of the true genotypes of the founders is $x_{m}^{f}$. The details to derive the equation shown above are available in the *Joint probability derivation* section in the Supplementary Methods. We assume that $P(x_{m}^{f})$ follows a discrete uniform probability for all possible combinations of the founder genotypes while omitting cases in which all founders have a same genotype.

### Emission probability

To obtain the emission probability $P(y_{mi}^{o}|h_{mi}^{o},x_{m}^{f})$, 3 additional probabilities are introduced: $P(x_{mi}^{\prime o}|x_{mi}^{o},e_{m}^{\mathrm{map}})$, $P(y_{mi}^{o}|x_{mi}^{\prime o},e^{\mathrm{seq}},w_{m})$, and $P(x_{mi}^{o}|h_{mi}^{o},x_{m}^{f})$. The first 2 probabilities incorporate the effect of read mismapping that would make true genotype unobservable. $P(x_{mi}^{\prime o}|x_{mi}^{o},e_{m}^{\mathrm{map}})$ is the probability that the observable genotype is $x_{mi}^{\prime o}$ when read mismapping occurred at marker *m* of *i*th offspring that has the true genotype $x_{mi}^{o}$. $P(y_{mi}^{o}|x_{mi}^{\prime o},e^{\mathrm{seq}},w_{m})$ represents the probability to observe the reads $y_{mi}^{o}$ when the observable genotype is $x_{mi}^{\prime o}$. $P(x_{mi}^{o}|h_{mi}^{o},x_{m}^{f})$ is the probability to observe the genotype $x_{mi}^{o}$ when the haplotype $h_{mi}^{o}$ was descendent from the founders having the genotype $x_{m}^{f}$. We then rewrite the emission probability as $l_{mi}^{o}\;=\;P(y_{mi}^{o}|h_{mi}^{o},x_{m}^{f},e_{m}^{\mathrm{map}},e^{\mathrm{seq}},w_{m})$, and it holds that


$$l_{mi}^{o}\;=\underset{x_{mi}^{o}}{\sum }\;P(y_{mi}^{o}|x_{mi}^{o},e_{m}^{\mathrm{map}},e^{\mathrm{seq}},w_{m})P(x_{mi}^{o}|h_{mi}^{o},x_{m}^{f}),$$

$$P(y_{mi}^{o}|x_{mi}^{o},e_{m}^{\mathrm{map}},e^{\mathrm{seq}},w_{m})\;=\underset{x_{mi}^{\prime o}}{\sum }\;P(y_{mi}^{o}|x_{mi}^{\prime o},e^{\mathrm{seq}},w_{m})P(x_{mi}^{\prime o}|x_{mi}^{o},e_{m}^{\mathrm{map}}),$$

where $P(x_{mi}^{o}|h_{mi}^{o},x_{m}^{f})$ equals 1 for the possible genotype under the constraint of the descendent haplotypes and founder genotypes in sample *i* and 0 for the other genotypes. For example, if sample 1 has $h_{1,1}^{o}\;=\;(h^{1}\;=\;1,h^{2}\;=\;4)$ at marker 1, indicating that the haplotypes descended from the first chromosome of founder 1 and the second chromosome of founder 2, and if the founder genotypes are $x_{1,1}^{f}\;=\;(0,0)$ and $x_{1,2}^{f}\;=\;(0,1)$, the genotype can only be heterozygotic . The parameter $e^{\mathrm{seq}}$ represents the global sequencing error rate that an unexpected allele read can be observed when the true genotype is either homozygote. We only assume that the sequencing error generates a read representing an opposite allele of the true allele. All markers are considered to have a same $e^{\mathrm{seq}}$ value. The parameters $w_{m}$ and $e_{m}^{\mathrm{map}}$ are the marker-specific allele read bias and mismapping rate at marker *m*. These are the key parameters that make GBScleanR differ from the other algorithms including magicImpute in which no allele read bias and a constant mismapping rate are assumed. $x_{mi}^{\prime o}$ indicates the observable genotype that results from accounting for the mismapping with $e_{m}^{\mathrm{map}}$. We assume that $e_{m}^{\mathrm{map}}$ takes 2 values $\left(e^{\mathrm{ref}},e^{\mathrm{alt}}\right)$. $e^{\mathrm{ref}}\;$ indicates the probability of incorrectly observing an alternative allele due to mismapped alternative reads when the true genotype is a reference homozygote. Similarly, $e^{\mathrm{alt}}$ represents the probability of incorrectly observing a reference allele due to mismapped reference reads when the true genotype is an alternative homozygote. In the calculation of $P(x_{mi}^{\prime o}|x_{mi}^{o},e_{m}^{\mathrm{map}})$, we do not consider the undercalling of heterozygotes and missing calls for which $P(y_{mi}^{o}|x_{mi}^{\prime o},e^{\mathrm{seq}},w_{m})$ accounts. Therefore, when the true genotype $x_{mi}^{o}$ is heterozygous, we can expect to observe that both alleles and an additional incorrect observation of either allele due to mismapping do not change the observable genotype $x_{mi}^{\prime o}$ that is heterozygous. On the other hand, for example, the incorrectly observed reference allele due to mismapping of reference reads at an alternative homozygous site makes the site can be observed as heterozygous. Thus, the probability $P(x_{mi}^{\prime o}|x_{mi}^{o},e_{m}^{\mathrm{map}}\;=\;(e^{\mathrm{ref}},e^{\mathrm{alt}}))$ takes the values listed in Table 1. The observed read counts $y_{mi}^{o}\;=\;\{y^{\mathrm{ref}},y^{\mathrm{alt}}\}$ follow binomial distribution


$$P(y_{mi}^{o}|x_{mi}^{\prime o}\;=\;0,e^{\mathrm{seq}},w_{m})\propto (1-e^{\mathrm{seq}}{)}^{y^{\mathrm{ref}}}(e^{\mathrm{seq}}{)}^{y^{\mathrm{alt}}},$$

$$P(y_{mi}^{o}|x_{mi}^{\prime o}\;=\;1,e^{\mathrm{seq}},w_{m})\propto (1-w_{m}{)}^{y^{\mathrm{ref}}}(w_{m}{)}^{y^{\mathrm{alt}}},$$

$$P(y_{mi}^{o}|x_{mi}^{\prime o}\;=\;2,e^{\mathrm{seq}},w_{m})\propto (e^{\mathrm{seq}}{)}^{y^{\mathrm{ref}}}(1-e^{\mathrm{seq}}{)}^{y^{\mathrm{alt}}},$$

given the observable genotype $x_{mi}^{\prime o}$. To reduce the negative impact of overrepresented mismapping reads that can make the probabilities of less probable genotypes being 0, we add 0.005 to the probabilities for the 3 possible genotypes if any of $x_{mi}^{\prime o}$ gave $P(y_{mi}^{o}|x_{mi}^{\prime o},e^{\mathrm{seq}},w_{m})\;=\;0$. The normalization constant for $P(y_{mi}^{o}|x_{mi}^{\prime o},e^{\mathrm{seq}},w_{m})$ is not shown in the equation above. Similarly, the emission probability for founders can be obtained by


$$l_{m}^{f}\propto \overset{N^{f}}{\underset{j}{\prod }}\;P(y_{mj}^{f}|x_{mj}^{f},e_{m}^{\mathrm{map}},e^{\mathrm{seq}},w_{m}),$$

where the normalization constant for $l_{m}^{f}$ is not shown. To omit the less probable founder genotypes, the probability $P(y_{mj}^{f}|x_{mj}^{f},e_{m}^{\mathrm{map}},e^{\mathrm{seq}},w_{m})$ is set to 0 if the result is <0.01.

**Table 1.** Probabilities associated with mismapping.

		Observable genotype $x_{mi}^{\prime o}$		
		0	1	2
True genotype $x_{mi}^{o}$	0	$\;1-e^{\mathrm{ref}}$	$e^{\mathrm{ref}}$	0
	1	0	1	0
	2	0	$e^{\mathrm{alt}}$	$1-e^{\mathrm{alt}}$

### Transition probability

The transition probability $P(h_{mi}^{o}|h_{m-1,i}^{o},x_{m-1}^{f})$ is used to describe the probability that a crossover occurs between adjacent markers. The sequence of the descendent haplotypes is modeled using a continuous-time Markov chain, as described previously (Zheng *et al.* 2014, 2015). The probability that the haplotype state transitions from $h_{m-1,i}^{o}$ to $h_{mi}^{o}$ between the markers can be obtained using


$$P(h_{mi}^{o}|h_{m-1,i}^{o})\;=\;e^{Q_{d_{m}}},m\neq 1,$$

where Q denotes the transition rate matrix that defines the rates at which a state transition occurs per unit of time. The matrix exponential is obtained by Higham's algorithm, which is implemented in the “expm” package of R (http://cran.r-project.org/web/packages/expm/index.html). To obtain transition rates that are proportional to the distances between adjacent markers, Q is multiplied by the genetic distance between markers *m* and *m* − 1 in Morgan *d_m_*. While our algorithm requires genetic distances between markers, genotype data obtained via NGS generally provide only information about the physical distances. Therefore, *d_m_* is calculated based on the physical distance and the expected genetic distance:


$$d_{m}\;=\;10^{-6}(\;p_{m}-p_{m-1})E^{d},m\neq 1,$$

where *p_m_* represents the physical position of marker *m* and E^d^ is the expected genetic distance per megabase pair that should be specified as the input parameter. For *m* = 1, the initial probability $P(h_{1i}^{o}|x_{1}^{f})$ is required and specified by the stationary distribution of the Markov process that can be obtained by normalizing the first eigenvector of the transposed transition probability matrix for a marker interval.

### Estimation of the best haplotype and genotype sequences

Together with the definitions described in the sections above, the joint probability to maximize can be rewritten as shown below:


$$P(H^{o},X^{f},Y^{o},Y^{f})\;=\underset{m=1}{\prod }\;l_{m}^{f}P(x_{m}^{f})\underset{i=1}{\prod }\;\left\{\underset{m=1}{\prod }\;l_{mi}^{o}\underset{m=2}{\prod }\;P(h_{mi}^{o}|h_{m-1,i}^{o},x_{m-1}^{f})P(h_{1i}^{o}|x_{1}^{f})\right\}$$

The Viterbi algorithm is used to maximize the joint probability and estimate the most likely haplotype sequences (Rabiner 1989). Considering that the sequence of founder genotypes itself does not follow the Markov process while the offspring haplotypes depend on the founder genotypes, the most likely founder genotypes at marker *m* can be estimated based on the scores (probabilities) and the states of the Viterbi trellises that were calculated for the offspring genotype from markers 1 to *m*. Therefore, our algorithm recursively calculates the Viterbi scores for offspring $v_{mi}^{o}(h_{mi}^{o}|x_{m}^{f})$ and founders $v_{m}^{f}(x_{m}^{f}|x_{m-1}^{f})$ and records the Viterbi paths for the sequences of offspring haplotypes $\psi_{mi}^{o}(h_{mi}^{o}|x_{m}^{f})$ and founder genotypes $\psi_{m}^{f}(x_{m}^{f})$, respectively. The recursive calculation of the integrated Viterbi algorithm proceeds as follows.

Initialization at *m* = 1:

The initialization starts with simply calculating the probability of having $h_{1i}^{o}$ when the combination of the founder genotypes $x_{1}^{f}$ is given.


$$v_{1i}^{o}(h_{1i}^{o}|x_{1}^{f})\;=\;l_{1i}^{o}P(h_{1i}^{o}|x_{1}^{f}),$$

where $P(h_{1i}^{o}|x_{1}^{f})$ is the initial probability that the *i*th offspring has the combination of haplotypes $h_{1i}^{o}\;=\;(h^{1},h^{2})$ at the first marker.

Recursion at 2≤m≤M:

The algorithm calculates the probabilities of state transitions from marker *m* − 1 to *m* as shown below:


$$\tau_{mi}(h_{mi}^{o}|h_{m-1,i}^{o},x_{m-1}^{f})\;=\;P(h_{mi}^{o}|h_{m-1,i}^{o})v_{m-1,i}^{o}(h_{m-1,i}^{o}|x_{m-1}^{f}),$$

$$\tau_{mi}^{\prime }(h_{mi}^{o}|x_{m}^{f},x_{m-1}^{f})\;=\;l_{mi}^{o}\underset{h_{m-1,i}^{o}}{\max}\;[\tau_{mi}(h_{mi}^{o}|h_{m-1,i}^{o},x_{m-1}^{f})],$$

where $\tau_{mi}(h_{mi}^{o}|h_{m-1,i}^{o},x_{m-1}^{f})$ is a matrix, each element of which indicates the probability of observing the transition from $h_{m-1,i}^{o}$ to $h_{mi}^{o}$ when $h_{m-1,i}^{o}$ and $x_{m-1}^{f}$ are given, while $\tau_{mi}^{\prime }(h_{mi}^{o}|x_{m}^{f},x_{m-1}^{f})$ indicates the probabilities of observing $h_{mi}^{o}$ when the most likely transition from $h_{m-1,i}^{o}$ to $h_{mi}^{o}$ occurs with given $x_{m}^{f}$ and $\;x_{m-1}^{f}.$

Then, calculate the Viterbi scores and the Viterbi paths for founders as


$$v_{m}^{f}(x_{m}^{f}|x_{m-1}^{f})\;=\;P(x_{m-1}^{f})l_{m-1}^{f}\underset{i}{\prod }\;\underset{h_{mi}^{o}}{\sum }\;\tau_{mi}^{\prime }(h_{mi}^{o}|x_{m}^{f},x_{m-1}^{f}),$$

$$\psi_{m}^{f}(x_{m}^{f}\;)\;=\arg\;\underset{x_{m-1}^{f}}{\max}\;[v_{m}^{f}(x_{m}^{f}|x_{m-1}^{f})].$$

Using $\psi_{m}^{f}(x_{m}^{f})$, which stores the most likely combination of founder genotypes at marker *m* − 1 when $x_{m}^{f}$ is given, we can obtain the Viterbi scores and the Viterbi paths for offspring as follows:


$$\psi_{mi}^{o}(h_{mi}^{o}|x_{m}^{f})\;=\arg\;\underset{h_{m-1,i}^{o}}{\max}\;[\tau_{mi}(h_{mi}^{o}|h_{m-1,i}^{o},x_{m-1}^{f}=\;\psi_{m}^{f}(x_{m}^{f}))],$$

$$v_{mi}^{o}(h_{mi}^{o}|x_{m}^{f})\;=\;\tau_{mi}^{\prime }(h_{mi}^{o}|x_{m}^{f},x_{m-1}^{f}=\;\psi_{m}^{f}(x_{m}^{f})).$$

Termination at *m* = *M*:

Finally, we obtain the probability of observing $x_{M}^{f}$ and the most likely combination of founder genotypes at marker *M* via the following calculation:


$$v_{M}^{\prime f}(x_{M}^{f})\;=\;P(x_{M}^{f})l_{M}^{f}\underset{i}{\prod }\;\underset{h_{mi}^{o}}{\sum }\;v_{Mi}^{o}(h_{Mi}^{o}|x_{M}^{f}),$$

$${\hat{x}}_{M}^{f}\;=\arg\;\underset{x_{M}^{f}}{\max}\;[v_{M}^{\prime f}(x_{M}^{f})].$$

Once ${\hat{x}}_{M}^{f}$ is determined, we can also obtain the most likely offspring haplotypes at marker *M*:


$${\hat{h}}_{Mi}^{o}\;=\arg\;\underset{h_{Mi}^{o}}{\max}\;[v_{Mi}^{o}(h_{Mi}^{o}|{\hat{x}}_{M}^{f})].$$

Backtracking from *M* − 1 to 1:

To retrieve the most likely sequences of founder genotypes and offspring haplotypes, we trace the Viterbi paths as shown below:


$${\hat{x}}_{m}^{f}\;=\;\psi_{m+1}^{f}({\hat{x}}_{m+1}^{f}),$$

$${\hat{h}}_{mi}^{o}\;=\;\psi_{m+1,i}^{o}({\hat{h}}_{m+1,i}^{o}|{\hat{x}}_{m+1}^{f}).$$

If multiple arguments have the same maximum score, one of them is randomly selected. To increase the accuracy, our algorithm executes 2 rounds of estimation: 1 from marker 1 to *M* and another in the reverse direction. The reverse direction round starts from the last marker *M* and uses the estimated founder genotypes ${\hat{x}}_{M}^{f}$ from the forward round as the only possible founder genotypes at marker *M*. The results from both rounds are then merged by combining the first half of the estimated genotypes $\left(m<\frac{M}{2}\right)$ from the reversed direction round with the last half from the forward direction round.

The estimated genotype at each marker in each sample ${\hat{x}}_{mi}^{o}$ can now be obtained based on ${\hat{h}}_{mi}^{o}$ and ${\hat{x}}_{m}^{f}$. To evaluate the reliability of the estimated genotypes, the marginal probability is also calculated:


$$P(x_{mi}^{o}|Y^{o},{\hat{X}}^{f})\;=\underset{{\hat{h}}_{mi}^{o}}{\sum }\;P(x_{mi}^{o}|h_{mi}^{o},{\hat{X}}^{f})P(h_{mi}^{o}|Y^{o},{\hat{X}}^{f}),$$

where $P(h_{mi}^{o}|Y^{o},{\hat{X}}^{f})$ can be obtained using the forward–backward algorithm (Rabiner 1989). The estimated genotype ${\hat{x}}_{mi}^{o}$ is set as missing if $P({\hat{x}}_{mi}^{o}|Y^{o},{\hat{X}}^{f})$ is smaller than a user specified threshold *P*_call_, with 0.9 as the default.

### Estimation of allele read biases and mismapping rates

GBScleanR requires 4 parameters E^d^, $e^{\mathrm{seq}}$, $e_{m}^{\mathrm{map}}$, and $w_{m}$. The expected genetic distance per megabase pair E^d^ and sequencing error rate $e^{\mathrm{seq}}$ take a single positive number that should arbitrarily be specified as input parameters, while the mismapping rates $e_{m}^{\mathrm{map}}$ and the allele read biases $w_{m}$ can be estimated via iterative parameter optimization (IPO). IPO is achieved by repeating the genotype estimation step and the parameter estimation step using the estimated genotype data. For the first cycle of the genotype estimation, $e_{m}^{\mathrm{map}}=(e^{\mathrm{ref}},e^{\mathrm{alt}})$ and $w_{m}$ are initialized to (0.005,0.005) and 0.5 for all markers, respectively. Thus, the first iteration does not consider marker-specific allele read biases and mismapping rates. The parameter estimation proceeds as described below. With the estimated genotypes from the first cycle, we can obtain the number of allele reads for each estimated genotype. Let $y_{mi}^{\mathrm{ref}}$ and $y_{mi}^{\mathrm{alt}}$ be the number of reference reads and alternative reads at marker *m* in sample *i*, including both offspring and founders. The allele read bias at marker *m* is estimated by the following:


$$w_{m}\;=\;\frac{E_{m}^{\mathrm{ref}}}{E_{m}^{\mathrm{ref}}+E_{m}^{\mathrm{alt}}},$$

$$E_{m}^{\mathrm{ref}}\;=\;\frac{r_{m}^{\mathrm{ref}}(0)+r_{m}^{\mathrm{ref}}(1)}{n_{m}(0)+n_{m}(1)},$$

$$E_{m}^{\mathrm{alt}}\;=\;\frac{r_{m}^{\mathrm{alt}}(2)+r_{m}^{\mathrm{alt}}(1)}{n_{m}(2)+n_{m}(1)},$$

$$r_{m}^{x}(g)\;=\underset{i}{\sum }\;[y_{mi}^{x}\delta (x_{mi}^{o}\;=\;g)],\;x\in \{\mathrm{ref},\;\mathrm{alt}\},$$

$$n_{m}(g)\;=\;2^{\delta (g\neq 1)}\underset{i}{\sum }\;[\delta (x_{mi}^{o}\;=\;g)],$$

where *g* is 0, 1, or 2 and indicates the genotype, $E_{m}^{\mathrm{ref}}$ represents the expected number of reference allele reads observed if 1 reference allele is on the chromosome, and $E_{m}^{\mathrm{alt}}$ represents the expected number of alternative allele reads if 1 alternative allele is on the chromosome. On the other hand, the mismapping rate at marker *m* can be obtained by calculating


$$e^{\mathrm{ref}}\;=\;\frac{\sum_{i}\;[\delta (P(y_{mi}^{o}|x_{mi}^{\prime o}\;=\;1,e^{\mathrm{seq}},w_{m})>0.99)\delta (x_{mi}^{o}\;=\;0)]}{\sum_{i}\;[\delta (x_{mi}^{o}\;=\;0)]},$$

$$e^{alt}\;=\;\frac{\sum_{i}\;[\delta (P(y_{mi}^{o}|x_{mi}^{\prime o}\;=\;1,e^{\mathrm{seq}},w_{m})>0.99)\delta (x_{mi}^{o}\;=\;2)]}{\sum_{i}\;[\delta (x_{mi}^{o}\;=\;2)]},$$

where δ is an indicator function that is equal to 1 if the argument is true and 0 if it is false. $P(y_{mi}^{o}|x_{mi}^{\prime o}\;=\;1,e^{\mathrm{seq}},w_{m})$ is the genotype probability to call a heterozygote and can be obtained as described in the *Emission probability* section. We counted the number of mismapping-induced erroneous genotype calls at which the genotype probability to be heterozygous based on the observed reads is more than 0.99 but the estimated genotype via the HMM is homozygous. The rates of those erroneous heterozygous calls at HMM-estimated homozygous calls are assumed to represent the mismapping rates at markers.

### Preparation of real data

As real data, we used the genotype data obtained from the *F*_2_ population that has been derived from a cross between *Oryza sativa* and *Oryza longistaminata* and reported in the previous study (Furuta *et al.* 2017). In brief, GBS was performed with a *Kpn*I–*Msp*I restriction enzyme pair using MiSeq with 75-bp paired-end sequencing and generated an average of 134,447±50,788 reads per sample. We obtained a total of 2,539,459 and 3,481,218 reads for *O. sativa* and *O. longistaminata* from the independent runs, respectively. The obtained reads were then processed via the TASSEL-GBS pipeline v2 by following the instructions in the manual with the default parameters (https://bitbucket.org/tasseladmin/tassel-5-source/wiki/Tassel5GBSv2Pipeline) (Glaubitz *et al.* 2014). The obtained SNP markers were then filtered to retain only those markers that were homozygous in each founder but biallelic between founders. Only the first SNP was selected if multiple SNPs were present within a 75-bp stretch. To filter out the erroneous genotype calls that were caused by overrepresented mismapping reads, read counts were set to 0 for genotype calls at which reads of either allele were mapped more than a threshold. The threshold was set at the 90th percentile of observed read counts at the obtained markers for each allele of each sample. Thus, this filtering was applied to each sample with the sample-specific thresholds for the reference and alternative reads. The resulting genotype data had 5,035 SNP markers for 814 *F*_2_ individuals with an average read depth of 0.85×.

### Generation of simulation data

The simulation data sets were generated by mimicking the real data of chromosome 7, which showed relatively severe biases (Supplementary Fig. 1). Each data set was therefore simulated to consist of 620 markers on a diploid chromosome with a physical length of 50 Mb and a genetic length of 2 Morgans. Genotype data sets were simulated for 3 scenarios: a 2-way *F*_2_ population from inbred founders (homoP2_F2), a 2-way *F*_1_ population from outbred founders (hetP2_F1), and 8-way recombinant inbred lines (RILs) from inbred founders (homoP8_RIL). For each scenario, we simulated populations that had 10, 100, and 1,000 individuals. The genotype of each individual was simulated as described in the *Genotype data simulation* section of the Supplementary Methods.

Based on the simulated genotype data, read counts were assigned to each marker of each individual, mimicking the allele read biases observed in the real data. The details are available in the *Read count data simulation* section of the Supplementary Methods. The expected offspring read depths were set to 0.1×, 0.25×, 0.5×, 0.75×, 1×, 2×, 3×, 10×, and 20×. The founder read depth was set to 5× for all data sets because founders are usually sequenced relatively deep even in low-coverage GBS studies. Since a typical genotyping pipeline filters out markers at which no read was observed in either of founders, we allocated at least 1 read to all markers of founders and then allocated the remaining reads (“nonzero” data sets). In addition, we also simulated data sets in which founders have no read at some markers and missing genotypes (“allowzero” data sets). Simulation data sets without assuming any allele read biases and mismapping reads were also generated with the same settings for the other parameters to generate genotype and read count data.

### Evaluation of algorithms

GBScleanR was tested and compared with LB-Impute and magicImpute. The input parameter settings are described in the *Input parameter* section of the Supplementary Methods. Each algorithm was evaluated by calculating the proportions of correctly estimated genotypes (correct call rate), incorrectly estimated genotypes (miscall rate), and missing genotype calls (missing call rate) for each offspring in the simulated data sets. The proportion of correct calls over nonmissing calls was also calculated as estimation accuracy. The means and standard deviations of these values were then obtained for each data set processed by each algorithm. The resultant scores are shown in Supplementary Data 1. To conduct an evaluation based on the concordance rate for the real data, we first selected relatively reliable genotype calls supported by more than 6 reads at markers that had less than a 20% missing rate and more than a 40% minor allele frequency. The read counts of the selected genotype calls were then set to 0. These masked real data were subjected to genotype estimation. The resultant estimated genotype data were evaluated based on the concordance rate, which is the proportion of estimated genotype calls matching the raw genotype calls at the masked genotype calls. Furthermore, we also calculated the averages of the missing rate, the number of recombinations, and the segregation distortion level in each chromosome. The segregation distortion level at each marker was obtained by dividing the chi-square value, which was calculated based on the assumption of a 1:2:1 genotype ratio for 3 possible genotypes, by the number of nonmissing genotype calls. The segregation distortion level of each chromosome was then obtained by summing up the segregation distortion levels of all markers in each chromosome. To identify recombination breakpoints of genome segments in the estimated genotype data, we searched the markers that showed genotypes switched between adjacent nonmissing markers. The middle points of those markers were considered recombination breakpoints.

We also compared the running time of the 3 algorithms using the “nonzero” simulation data sets in the homoP2_F2, hetP2_F1, and homoP8_RIL scenarios. GBScleanR that has been implemented in R requires loading genotype data into the R environment and executing functions for genotype estimation step by step, while magicImpute and LB-Impute that were written in Mathematica and Java directly process input files and generate output files. Therefore, the overall process of GBScleanR including data loading, mating design specification, genotype estimation, and output data generation was measured as the running time of GBScleanR. The running time of magicImpute and LB-Impute was measured from the start to the end of the process. All computation was executed on a workstation with an AMD Ryzen Threadripper 2990WX CPU having 32 cores/64 threads and 64 Gb of RAM. Since magicImpute automatically tries to use half the number of threads for parallel computation, we also let GBScleanR use 32 threads for a fair comparison of running time. As LB-Impute does not support parallel computation and the main target of the running time comparison was GBScleanR versus magicImpute, LB-Impute was executed on a single thread.

## Results

### Evaluation using simulation data sets

**Fig. 1.**
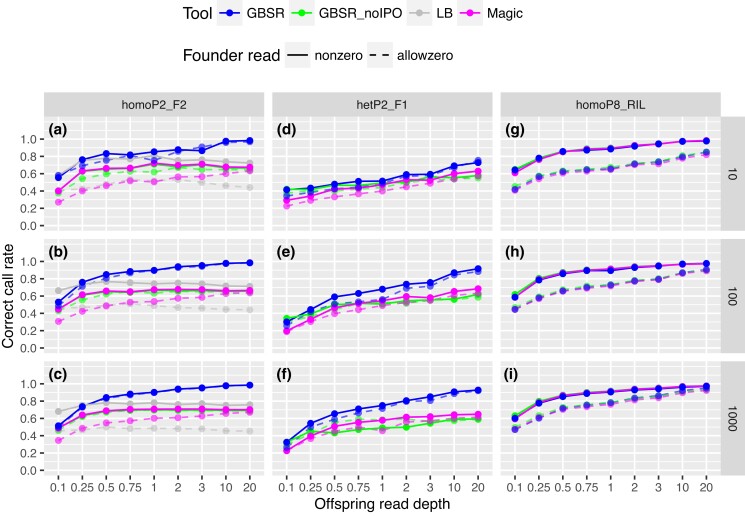
Offspring genotype estimation accuracies for the simulation data. The plots show correct call rates for the data sets with given read depths for offspring (*x*-axis) in the homoP2_F2 (a–c), hetP2_F1 (d–f), and homoP8_RIL (g–i) scenarios. The rows of the panels indicate the differences in the number of samples in the simulated data, as shown in the strips on the right. Solid lines and dashed lines represent the results for the data sets with (allowzero) or without (nonzero) missing founder reads. “GBSR” and “GBSR_noIPO” represent GBScleanR with and without IPO. “LB” and “Magic” indicate LB-Impute and magicImpute, respectively.

We first evaluated the 3 algorithms using the simulation data sets in the homoP2_F2, hetP2_F1, and homoP8_RIL scenarios (Fig. 1 and Supplementary Data 1). The superiority of GBScleanR lies in the IPO in which allele read biases and mismapping rates of markers are iteratively estimated. Thus, we also tested GBScleanR without IPO to demonstrate the effect of IPO on genotype estimation. In the homoP2_F2 scenario, GBScleanR with IPO scored the best correct call rates for almost all data sets (Fig. 1a–c). When the simulation data sets were generated without allowance to have no read at any markers in founders (indicated as “nonzero” in Fig. 1), LB-Impute showed the highest correct call rates for the data sets with a 0.1× offspring read depth. However, LB-Impute also scored the highest miscall rates for those data sets (Supplementary Fig. 3a–c). These increases in miscall rates resulted in the reduction of the estimation accuracy that was measured as the proportion of correct calls over nonmissing calls (Supplementary Fig. 4a–c). A reduction in accuracy was observed in all of the tested algorithms when no reads at some markers were allowed for founders (shown as “allowzero” in Fig. 1 and Supplementary Fig. 4). Nevertheless, GBScleanR was less affected by the markers with 0 reads in founders and outperformed the other algorithms. In addition, GBScleanR with IPO only increased the correct call rate as offspring read depth increased, while the other algorithms showed a plateau at 0.5–20× in the results for the “nonzero” data sets (Fig. 1a–c). The difference in the correct call rate between GBScleanR with IPO and the other algorithms reached more than 25 percentage points at maximum in the data with a 20× offspring read depth. On the other hand, the algorithms except for GBScleanR with IPO showed an increase in the miscall rate as the read depth increased (Supplementary Fig. 3a–c). The increase in the miscall rate resulted in the reduction of the estimation accuracy as the read depth increased (Supplementary Fig. 4a–c).

Similar to the results in homoP2_F2, GBScleanR showed the best correct call rates in almost all data sets of hetP2_F1, and the difference between the algorithms reached more than 25 percentage points at maximum (Fig. 1d–f and Supplementary Fig. 4d–f). Unlike the homoP2_F2 data sets, the genotype estimation accuracy for the hetP2_F1 data sets was highly affected by the number of samples. The increase in the correct call rate between the data sets with 10 and 1,000 samples reached ∼26 percentage points at maximum in the hetP2_F1 data sets with a 3× offspring read depth, whereas the maximum increase in the score in the homoP2_F2 data sets was ∼14 percentage points (Supplementary Data 1).

Unlike the F2 data sets, we could not find any remarkable difference in the accuracy of genotype estimation for the homoP8_RIL data sets (Fig. 1g–i and Supplementary Fig. 4g–i). This result seems to have reflected the higher homozygosity in the simulated genotypes. Since the offspring in the homoP8_RIL data sets had homozygotes at almost all markers due to repeated self-fertilization, only the reads of either allele representing the true genotype could be observed at any markers.

To compare the performance of the algorithms in the cases where no allele read bias and mismapping exist in data, we simulated data sets without assuming allele read bias and mismapping for generating read count data. All 3 algorithms scored almost the same estimation accuracy for the nonerror data sets and did not show any reduction of the accuracy even if read depth increased in any scenarios (Supplementary Fig. 5 and Data 2).

GBScleanR and magicImpute have been designed to simultaneously estimate the genotypes of both founders and offspring. In addition, both algorithms provide phasing information for the offspring genotypes. As expected from the estimation accuracy for offspring, GBScleanR also outperformed magicImpute in the estimation of founder genotypes and phased offspring genotypes (Supplementary Figs. 6 and 7). The details were described in the Supplementary Results.

### Less biased estimation by GBScleanR

**Fig. 2.**
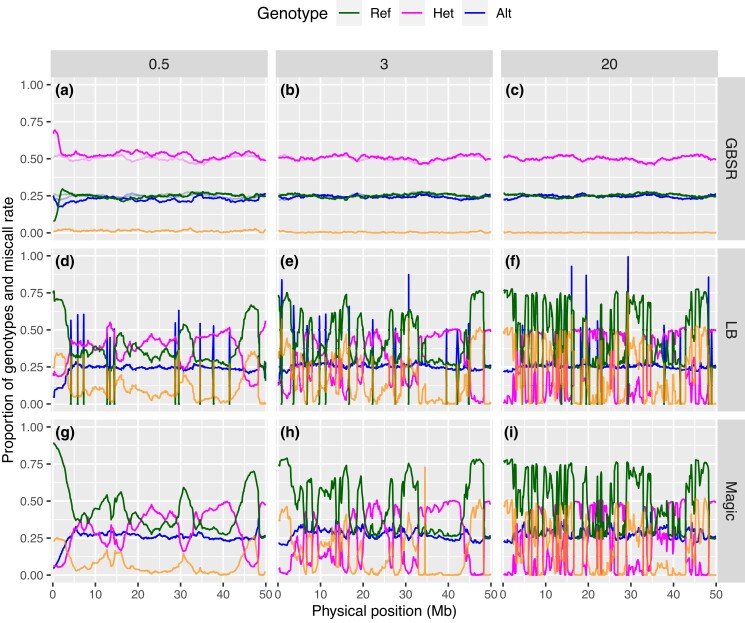
Genotype ratios at markers ordered along a simulated chromosome. The plots show genotype ratios calculated from the estimated genotype data for the homoP2_F2 data set with 1,000 offspring and 620 markers with offspring read depths at 0.5×, 3×, and 20× without allowance of no reads in founders. “GBSR” (a–c), “LB” (d–f), and “Magic” (g–i) indicate genotypes estimated by GBScleanR, LB-Impute, and magicImpute, respectively. Proportions of reference homozygous, heterozygous, and alternative homozygous genotypes are represented by green, magenta, and blue lines, respectively. Orange lines indicate miscall rates at markers. True genotype ratios are indicated by transparent lines in the panels for GBScleanR (a–c).

We further evaluated the quality of the estimated genotypes by visualizing the estimated genotype ratio at each marker. This visual inspection employed the “nonzero” homoP2_F2 data sets that have 1,000 offspring with offspring read depths at 0.5×, 3×, and 20×. In an *F*_2_ population derived from inbred parents, we can expect a 1:2:1 genotype ratio in offspring if no segregation distortion occurs. As shown in Fig. 2, the estimations by GBScleanR showed 1:2:1 segregation at almost all markers, which nearly completely overlapped with the true genotype ratio throughout the chromosome, except for the data set with 0.5× offspring reads (Fig. 2a–c). In addition, miscalls were distributed almost uniformly throughout the entire chromosome, with a very small amount per marker. In contrast, LB-Impute showed severely biased genotype ratios with high miscall rates clustered at several loci on the chromosome (Fig. 2d–f). The increase in offspring read depth made biased genotype estimation more severe and increased miscall rates, as also shown in Supplementary Fig. 3. Similar results were observed in the case of magicImpute (Fig. 2g–i). Even though fewer miscalls were generated in magicImpute compared to LB-Impute, many regions showed a biased estimation of reference homozygotes particularly in the data set with 0.5× offspring read depth (Fig. 2g). The loci showing higher miscall rates overlapped between the plots for LB-Impute and magicImpute. Thus, these miscalls and biased genotype estimations might be caused by error-prone markers. Similar results were observed in the data sets with 10 and 100 offspring (Supplementary Figs. 8 and 9). Although the true genotypes did not show a 1:2:1 ratio due to the small population size in the data sets with 10 offspring, GBScleanR showed the estimated genotype ratios that highly overlapped with the true ratios (Supplementary Fig. 8a–c). These results demonstrated the robustness of genotype estimation by GBScleanR even at error-prone markers.

### Evaluation using real data

**Fig. 3.**
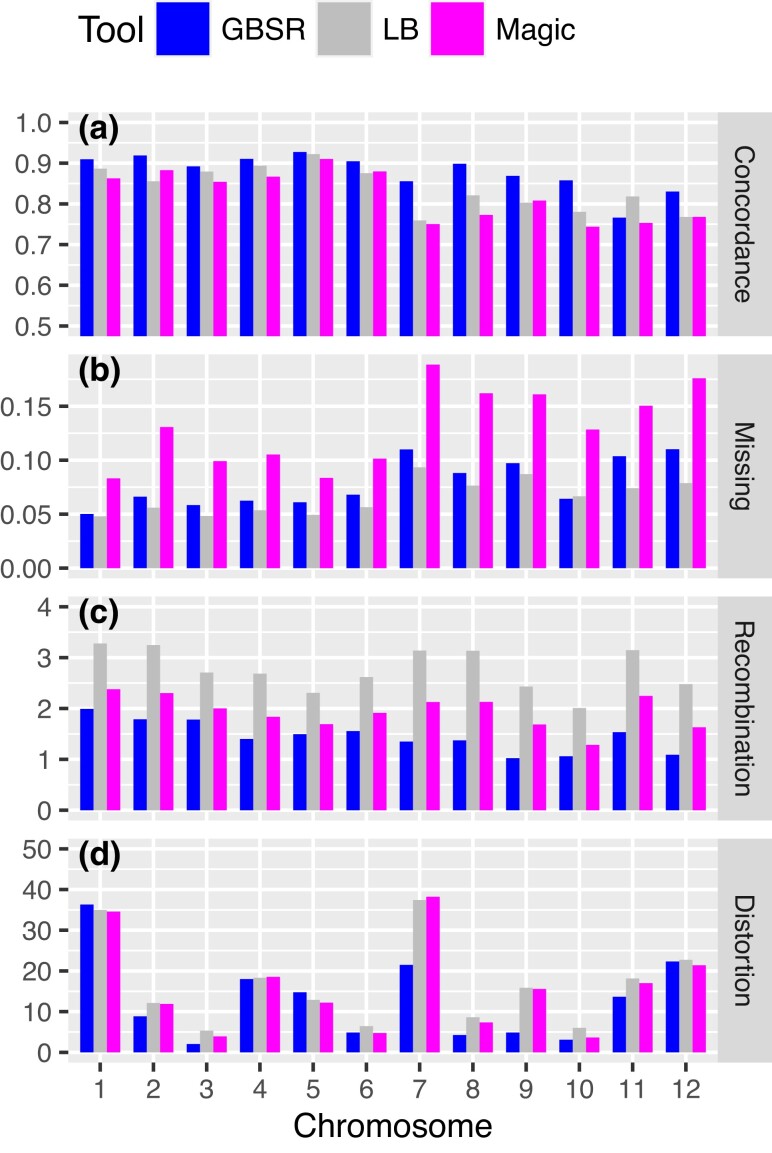
Evaluation of genotype estimation accuracy using the real data. a) Concordance rates of true genotype calls and estimated genotype calls at masked genotype calls are shown for each chromosome. The averages of the missing rate (b), the number of recombinations (c), and the segregation distortion level (d) are also visualized as bar plots for each chromosome. Blue, gray, and magenta bars indicate the values of GBScleanR (GBSR), LB-Impute (LB), and magicImpute (Magic), respectively.

We finally evaluated the algorithms using real data containing error-prone markers. First, we measured the concordance rate of the estimated genotype calls using the masked genotype data created from the real data. GBScleanR scored the best concordance rate in all chromosomes except for chromosome 11 in comparison with LB-Impute and magicImpute (Fig. 3a and Supplementary Data 5). Relatively large differences were observed in chromosomes 7, 8, and 10, in which GBScleanR scored 10.5, 12.5, and 12.0 percentage points higher concordance rates than magicImpute, respectively. On the other hand, chromosome 5 showed only a 1.7 percentage point difference between GBScleanR and magicImpute. Relatively low concordance rates were observed in the chromosomes with more severe allele read biases, which were chromosomes 7–12, than those in the other chromosomes (Fig. 3a and Supplementary Fig. 1).

Even though GBScleanR showed better concordance rates than the others, the genotype concordance does not directly mean higher accuracy because we cannot know the true genotype of the real data, and there is no evidence of whether the genotype calls selected for masking were truly reliable or contained a large number of erroneous genotype calls. To further evaluate the performance of the algorithms, we then calculated the average missing rate and the number of recombinations in each chromosome. In the case of the missing rate, the highest values were scored by magicImpute for all chromosomes, while GBScleanR and LB-Impute showed similar scores in a majority of chromosomes (Fig. 3b and Supplementary Data 5). Similar to the concordance rate, chromosomes with more severe allele read biases showed higher missing rates. A clear difference between the algorithms was observed in the number of recombinations (Fig. 3c and Supplementary Data 5). LB-Impute always estimated the most frequent recombinations, followed by magicImpute, whereas GBScleanR showed the fewest numbers of recombinations on all chromosomes. To further evaluate the recombination events, we measured the genomic segment lengths between the recombination breakpoints in chromosomes (Table 2 and Supplementary Data 6). We found that LB-Impute estimated many more short segments. The genotype data estimated by GBScleanR had 116 and 430 segments in the ranges of 0–1 and 1–2 Mb, respectively (Table 2). On the other hand, LB-Impute estimated the genotype data with 2,164 and 4,166 segments ranging 0-1 and 1-2 Mb, respectively. The magicImpute algorithm generated fewer short segments than LB-Impute but a larger number than GBScleanR, which were 627 and 1,716, respectively. In addition, GBScleanR, magicImpute, and LB-Impute estimated 14, 235, and 948 segments ranging 0-1 Mb that were heterozygous and flanked by the same homozygous genotypes at both sides, which indicated double crossovers within 1-Mb stretches (Table 2). The probability of a double crossover within 1 Mb is ∼0.08% when 1 Mb equals 4 cM in an *F*_2_ population. Thus, the expected number of double crossovers is < 2 in the population with 814 individuals derived from 1,628 gametes. Even though the 14 double crossovers within 1-Mb stretches generated by GBScleanR were too frequent for an ideal *F*_2_ population, considering the distant cross to generate the given population, these results suggested that GBScleanR estimated more probable and acceptable numbers of recombinations. The excess crossovers over short distances seemed to artificially push up the numbers of recombinations in the estimated genotype data of LB-Impute and magicImpute.

**Table 2.** The number of genome segments.

		Segment length	
		0–1 Mb	1–2 Mb
Total*^a^*	GBScleanR	116	430
	magicImpute	627	1,716
	LB-Impute	2,164	4,166
Double crossovers*^b^*	GBScleanR	14	40
	magicImpute	235	558
	LB-Impute	948	1,395

a The total number of genome segments that have a same genotype continuously with the length of 0–1 or 1–2 Mb.

b The number of genome segments that have a heterozygous genotype continuously with the length of 0–1 or 1–2 Mb and flanked by homozygous segments of a same allele.

**Fig. 4.**
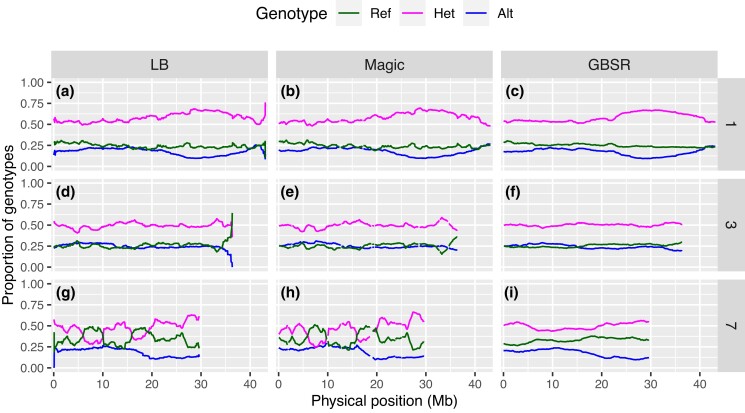
Genotype ratio of estimated offspring genotypes in the real data. Genotype ratios at markers along chromosomes 1 (a–c), 3 (d–f), and 7 (g–i) in the real data are shown as line plots. “LB,” “Magic,” and “GBSR” indicate LB-Impute, magicImpute, and GBScleanR, respectively. Proportions of the reference homozygous, heterozygous, and alternative homozygous genotypes are represented by green, magenta, and blue lines, respectively.

Finally, we measured the segregation distortion level (Fig. 3d and Supplementary Data 5). A relatively low distortion level was observed in the genotype data estimated via GBScleanR compared with those estimated by LB-Impute and magicImpute (Fig. 3d). Chromosome 1 was the most distorted in the genotype data estimated by GBScleanR, while the other algorithms also showed similar distortion levels in this chromosome. On the other hand, the highest distortion level was observed in chromosome 7 in the cases of LB-Impute and magicImpute (Fig. 3d). Unlike chromosome 1, GBScleanR showed a much smaller distortion level that was approximately half of the levels scored by the other algorithms. In contrast, the 3 algorithms all resulted in the mildest distortion in chromosome 3 (Fig. 3d). To visually inspect the segregation distortion levels, we created line plots of the estimated genotype ratios for chromosomes 1, 3, and 7 (Fig. 4). The line plots indicated that all of the algorithms estimated quite similar genotypes for chromosomes 1 and 3, while clearer differences were observed in the plots for chromosome 7. As fewer markers on chromosomes 1 and 3 showed severe allele read biases, these chromosomes might have fewer error-prone markers (Supplementary Fig. 1). Therefore, all of the algorithms resulted in similar estimations (Fig. 4a–f). Since all algorithms showed similar distortion patterns around 30 Mb of chromosome 1, the distortion on chromosome 1 was likely to be the true genetic characteristic of this population but not due to the misestimation (Fig. 4a–c). On the other hand, slight distortions at 5, 10, and 35 Mb of chromosome 3 that were observed in the genotypes estimated by LB-Impute and magicImpute, but not by GBScleanR, might be artifacts produced by biased estimations at error-prone markers (Fig. 4d–f). In the case of chromosome 7, LB-Impute and magiImpute obviously failed to estimate the genotypes and produced artificially distorted genotype ratio patterns (Fig. 4g–i). In contrast, GBScleanR showed a smooth and less fluctuating change in the genotype ratio, which was an acceptable pattern as the genotype ratio of an *F*_2_ population, throughout the chromosome, although severe distortion was observed, probably due to the true segregation distortion (Fig. 4i). The other chromosomes also exhibited similar results to those observed for chromosomes 1, 3, and 7 (Supplementary Fig. 10). LB-Impute and magicImpute always resulted in highly biased genotype estimations at many loci and fluctuated genotype ratios throughout the chromosomes. In clear contrast to those results, GBScleanR showed relatively smooth and gradual changes in the genotype ratios throughout the chromosomes. Even though some loci showed distorted genotype ratios, the majority of chromosomal regions had ratios relatively close to 1:2:1 in almost all chromosomes in the GBScleanR results compared with those of the other algorithms. Overall, the performance evaluation on the real data demonstrated the superiority and robustness of GBScleanR against error-prone markers.

### Effect of the iterative optimization for allele read biases and mismapping rates

In addition to demonstrating the superiority of GBScleanR, we tested the effect of IPO on genotype estimation by switching on and off the iterative calculations for either or both of allele read biases and mismapping rates. As already shown, GBScleanR with no IPO (noIPO) led to a reduction in the estimation accuracy for the simulation data sets of homoP2_F2 and hetP2_F1 compared with GBScleanR with IPO (withIPO) (Supplementary Fig. 1^1^). On the other hand, when only the iterative optimization for the marker-specific mismapping rate was turned off (fixMismap), no remarkable difference was observed. Interestingly, only turning off the iterative optimization of marker-specific allele read bias (fixBias) resulted in even worse accuracy than noIPO. The reduction of accuracy by the fixBias setting might be caused by misestimation of the mismapping rates that were inferred based on genotype data containing miscalls generated due to the assumption of the fixed allele read bias. Similar to the comparison between GBScleanR and magicImpute, the homoP8_RIL showed no remarkable difference even with or without IPO (Supplementary Fig. 1^1^).

Unlike the cases in the simulation data sets, the real data showed that not only the fixBias and noIPO settings but also the fixMismap setting might result in less accurate estimation (Supplementary Fig. 1^2^ and Data 7). Although we cannot know the true genotype of the real data, the fixMismap showed lesser smooth lines in the plots and more severe genotype distortions than the withIPO setting (Supplementary Fig. 1^2^). All of the metrics including the concordance rate and the recombination rate indicated the superiority of the withIPO setting over the other settings (Supplementary Data 7). These results indicated that the real data may have a nonrandom pattern of the mismapping-prone marker distribution, and the iterative optimizations for both allele read biases and mismapping rates are required for proper genotype estimation. GBScleanR with IPO showed robustness against both types of errors.

### Comparing running time

We also compared running times using the simulation data sets. As shown in Table 3, GBScleanR completed all processes of genotype estimation faster than LB-Impute and magicImpute for the simulation data sets of biparental populations, including homoP2_F2 and hetP2_F1. In the case of the largest homoP2_F2 data set that consists of 1,000 samples, GBScleanR took only 17 s even with 4 cycles of IPO, while magicImpute and LB-Impute consumed 6.4 and 122.7 times as long. On the other hand, a 2.1-times longer calculation at the maximum was observed in GBScleanR with 4 cycles of IPO compared to magicImpute for the homoP8_RIL data sets (Table 3). Nevertheless, IPO was not required for RIL populations, as shown in Fig. 1, and GBScleanR without IPO could finish the calculations 48% faster than magicImpute even in the homoP8_RIL data set with 1,000 samples.

**Table 3.** Running time.

Data set	No. of samples	GBScleanR*^a^*	magicImpute	LB-Impute
homoP2_F2	10	2 (1)	3	23
homoP2_F2	100	3 (1)	14	211
homoP2_F2	1,000	17 (8)	108	2,086
hetP2_F1	10	4 (2)	4	NA
hetP2_F1	100	11 (4)	20	NA
hetP2_F1	1,000	81 (24)	170	NA
homoP8_RIL	10	36 (9)	98	NA
homoP8_RIL	100	205 (50)	336	NA
homoP8_RIL	1,000	1,736 (432)	829	NA

Running times in seconds for each data set are shown.

a The numbers in parenthesis indicate the running times of GBScleanR without the IPO.

## Discussion

GBScleanR is an R package and consists of a main tool that conducts error correction and utility functions that handle genotype data in the variant call format (VCF). Supplementary Fig. 13 presents the data analysis workflow and the related functions that are implemented in GBScleanR. The algorithm is designed to estimate the true genotype calls along chromosomes from the given allele read counts in a VCF file that is generated by SNP callers such as GATK and TASSEL-GBS (McKenna *et al.* 2010; Glaubitz *et al.* 2014). The current implementation supports genotypic data for mapping populations that are derived from 2 or more diploid founders followed by selfings, sibling crosses, or random crosses, for example, *F*_2_- and 8-way RILs. Our method assumes that markers are biallelic and ordered along the chromosomes and the reads are mapped onto a reference genome sequence. To access the large amounts of genotype data required, an input VCF file is first converted into a genomic data structure (GDS) file (Zheng *et al.* 2012). This conversion automatically filters out nonbiallelic markers. GBScleanR provides functions for data visualization, filtering, and loading/writing a VCF file. Furthermore, the data structure of the GDS file created via this package is compatible with that used in the SeqArray package and can be further converted to a derivative GDS format used in the SNPRelate, GWASTools, and GENESIS packages, which are designed to handle large amounts of variant data and conduct regression analysis (Gogarten *et al.* 2012, 2019; Zheng *et al.* 2012, 2017). Therefore, GBScleanR can be built in a pipeline for a genetic study that allows us smooth access to the given large genotype data for visualization, filtering, error correction, and regression analysis without the requirement for extra data format conversion steps.

The present study introduced an algorithm to provide robust error correction on genotype data with error-prone markers. Any genotype data derived from GBS or other RRS-based genotyping techniques have the chance to contain such error-prone markers even after trying to filter out erroneous markers. Particularly, a population derived from a cross between distant relatives, as in our case in which a cultivated rice *O. sativa* and a wild rice *O. longistaminata* were crossed, may potentially have a relatively high proportion of error-prone markers. If a model uniformly assumes a 50% chance of obtaining a read for either allele for all markers at a heterozygous site, any HMM-based methods and sliding window methods can theoretically be deceived by the error-prone markers. In addition, erroneous genotype calls induced by mismapping easily mess up genotype data and lead to unexpected detection of heterozygous genotypes and overrepresented recombination events. Since we could not distinguish which reads were mismapped reads in the real data and had no clue to estimate the true pattern of mismappings, we assumed a random distribution of mismapping-prone markers in the data simulation. Those randomly distributed mismapping-prone markers did not show any remarkable effect on the estimation accuracy even with the fixMismap setting (Supplementary Fig. 1^1^). On the other hand, mimicked marker-specific allele read biases caused the reduction in the estimation accuracy with the fixBias and noIPO settings (Supplementary Fig. 1^1^). However, the fixMismap setting resulted in less reliable genotype estimation than the withIPO setting for the real data (Supplementary Fig. 1^2^ and Data 7). These results indicate that real data seem to have a specific pattern of error-prone marker distribution that interferes with proper genotype estimation. The distribution of error-prone markers would be different between given populations and affected by actual biological variations that exist in given genomes. Our study indicated the importance to incorporate marker-specific error patterns in the HMM for accurate genotype estimation and demonstrated the superiority and robustness of GBScleanR. The distortion level and the recombination frequency have large effects on downstream genetic analyses, and those values themselves are research targets. Any genetic and genomic study relies on the given genotype data, and the accuracy of genotyping has a crucial effect on the quality of such research. The accuracy of genotyping can be evaluated by 3 criteria: correct calls, miscalls, and missing calls. Usually, miscalls have a more destructive effect on post-genotyping analyses than missing calls because post-genotyping analyses regularly suppose all genotype calls to be true even if those include miscalls and potentially generate misleading results. Furthermore, if the wrongly genotyped markers are clustered in a particular region, all statistical calculations for that region will be unreliable. Our study also demonstrated the reliability of GBScleanR as a support tool for genetic studies that handle genotype data even with or without error-prone markers.

In this study, we conducted filtering based on a quantile of read counts for the real data to filter out genotype calls supported by overrepresented sequences that are possibly derived from paralogous and repetitive sequences. A large number of overrepresented mismapped reads do not increase the reliability of genotype calls but rather cause misestimation of homozygotes as heterozygotes. Although GBScleanR tries to detect mismapped reads, we recommend filtering out overrepresented reads to reduce their negative impact on estimation. Nevertheless, GBScleanR accepts error-prone markers and recognizes the error pattern via IPO. Therefore, no strict filtering based on allele frequency and the missing rate is required, which is regularly performed for RRS-based genotyping data (Kim *et al.* 2016; Scheben *et al.* 2017). These filtering steps usually remove a large number of the detected SNP markers (Gardner *et al.* 2014; Pootakham *et al.* 2015; Celik *et al.* 2016; Boyles *et al.* 2017). In the case of our real data, filtering to remove possible error-prone markers based on, for example, minor allele frequency <5% and missing rate >20%, left only 162 out of 5,035 SNPs. Markers that would be filtered out may contain partial information about the true genotypes. GBScleanR can integrate those less informative markers into the error correction process by tweaking the HMM. Since there is no clear guideline for filtering strength, which should differ depending on the data set used, filtering criteria are left in the hands of researchers. GBScleanR allows users to skip such an arbitrary step and improves the cost efficiency of the RRS-based genotyping system by reducing the number of unused reads obtained via costly NGS.

As GBScleanR requires the SNP markers ordered along chromosomes, reference genome sequences are a fundamental resource. The recent remarkable development of the third-generation sequencers, named Sequel by Pacific Bioscience and MinION by Oxford Nanopore Technologies, enables us to obtain high-quality genome sequences at a reasonable cost (Rhoads and Au 2015; Lu *et al.* 2016). Reference genome information for any organism is expected to become available soon. Thus, RRS-based genotyping systems underpinned by a flexible robust error correction tool such as GBScleanR will become practical platforms for use in breeding programs requiring a prompt and efficient scheme under limited periods and budgets. Distant relatives, including the ancestral wild species of domesticated species, are important genetic resources, particularly in plant breeding, that can improve abiotic and biotic stress resistance (Atwell *et al.* 2014; Kole *et al.* 2015; Brozynska *et al.* 2016). For such genetic studies and breeding projects using wild species, GBScleanR could be the first choice as a fundamental tool for genotype identification coupled with an RRS-based genotyping system.

## Supplementary Material

iyad055_Supplementary_Data

## Data Availability

The stable version of GBScleanR is available on Bioconductor (https://bioconductor.org/packages/GBScleanR/). The latest developmental version is available on GitHub (https://github.com/tomoyukif/GBScleanR). This package and the source code are distributed under GNU General Public License version 3. The vcf file of the real data and R scripts for the data analyses performed in this study are also available on GitHub (https://github.com/tomoyukif/GBSR_SupFiles). Further description of the sequencing reads for the real data can be found in Furuta *et al.* (2017). Supplemental material available at *GENETICS* online.
