# Supplementary material for: GBScleanR: robust genotyping error correction using a hidden Markov model with error pattern recognition
Source: Genetics. 2023 Mar 29;224(2):iyad055. doi: 10.1093/genetics/iyad055 (PMC10213493; doi:10.1093/genetics/iyad055)
Supplement: iyad055_Supplementary_Data [file iyad055_supplementary_data.zip › SupplementaryMaterials.pdf]

Supplementary Materials for

GBScleanR: robust genotyping error correction using a hidden Markov model with error pattern recognition.

Tomoyuki Furuta[1,*], Toshio Yamamoto[1], and Motoyuki Ashikari[2]

[1] Institute of Plant Sci. and Resources, Okayama University, Chu-oh 2-20-1, Kurashiki, Okayama, 710-0046, Japan

[2] Bioscience and biotechnology center, Nagoya University, Furo-cho, Chikusa, Nagoya, Aichi, 464-8601, Japan

[*] Corresponding author

Tel: +81-86-434-1208;

Email: f.tomoyuki@okayama-u.ac.jp

Supplementary Methods

Supplementary Results

Supplementary Table 1

Supplementary Figures 1-13

Supplementary Methods

Joint probability derivation

The joint probability $P(\boldsymbol{H}^{\mathrm{o}}, \boldsymbol{X}^{\mathrm{f}}, \boldsymbol{Y}^{\mathrm{o}}, \boldsymbol{Y}^{\mathrm{f}})$ can be transformed as seen below.

$$P(\boldsymbol{H}^{\mathrm{o}}, \boldsymbol{X}^{\mathrm{f}}, \boldsymbol{Y}^{\mathrm{o}}, \boldsymbol{Y}^{\mathrm{f}}) = P(\boldsymbol{H}^{\mathrm{o}}, \boldsymbol{Y}^{\mathrm{o}} | \boldsymbol{X}^{\mathrm{f}}, \boldsymbol{Y}^{\mathrm{f}}) P(\boldsymbol{X}^{\mathrm{f}}, \boldsymbol{Y}^{\mathrm{f}})$$

Each probability on the right hand of the equation can be farther transformed.

$$P(\boldsymbol{H}^{\mathrm{o}}, \boldsymbol{Y}^{\mathrm{o}} | \boldsymbol{X}^{\mathrm{f}}, \boldsymbol{Y}^{\mathrm{f}}) = P(\boldsymbol{Y}^{\mathrm{o}} | \boldsymbol{H}^{\mathrm{o}}, \boldsymbol{X}^{\mathrm{f}}) P(\boldsymbol{H}^{\mathrm{o}}, \boldsymbol{X}^{\mathrm{f}})$$

$$P(\boldsymbol{X}^{\mathrm{f}}, \boldsymbol{Y}^{\mathrm{f}}) = P(\boldsymbol{Y}^{\mathrm{f}} | \boldsymbol{X}^{\mathrm{f}}) P(\boldsymbol{X}^{\mathrm{f}})$$

We assume the independence of the offspring and then rewrite the first equation as

$$P(\boldsymbol{H}^{\mathrm{o}}, \boldsymbol{Y}^{\mathrm{o}} | \boldsymbol{X}^{\mathrm{f}}, \boldsymbol{Y}^{\mathrm{f}}) = \prod_{i=1}^{N^{\mathrm{o}}} P\left(y_i^{\mathrm{o}} | h_i^{\mathrm{o}}, \boldsymbol{X}^{\mathrm{f}}\right) P\left(h_i^{\mathrm{o}} | \boldsymbol{X}^{\mathrm{f}}\right).$$

We can now treat $P(y_i^{\mathrm{o}} | h_i^{\mathrm{o}}, \boldsymbol{X}^{\mathrm{f}}) P(h_i^{\mathrm{o}} | \boldsymbol{X}^{\mathrm{f}})$ as the HMM for i^{th} offspring's haplotypes and the read counts, given the founder genotype $\boldsymbol{X}^{\mathrm{f}}$. $P(\boldsymbol{Y}^{\mathrm{f}} | \boldsymbol{X}^{\mathrm{f}}) P(\boldsymbol{X}^{\mathrm{f}})$ is the product of the probability of observing $\boldsymbol{Y}^{\mathrm{f}}$ under the condition $\boldsymbol{X}^{\mathrm{f}}$ and the probability of obtaining $\boldsymbol{X}^{\mathrm{f}}$. The founder genotypes at each marker can be considered independent of each other and do not follow a Markov process. Therefore, our algorithm tries to maximize the following joint probability:

$$P(\boldsymbol{H}^{\mathrm{o}}, \boldsymbol{X}^{\mathrm{f}}, \boldsymbol{Y}^{\mathrm{o}}, \boldsymbol{Y}^{\mathrm{f}}) = \prod_{i=1}^{N^{\mathrm{o}}} \left\{ \prod_{m=1}^{M} P\left(y_{mi}^{\mathrm{o}} | h_{mi}^{\mathrm{o}}, \boldsymbol{x}_m^{\mathrm{f}}\right) \prod_{m=2}^{M} P\left(h_{mi}^{\mathrm{o}} | h_{m-1,i}^{\mathrm{o}}, \boldsymbol{x}_{m-1}^{\mathrm{f}}\right) P\left(h_{1i}^{\mathrm{o}} | \boldsymbol{x}_1^{\mathrm{f}}\right) \right\}$$

$$\times \prod_{m=1}^{M} P\left(\boldsymbol{y}_m^{\mathrm{f}} | \boldsymbol{x}_m^{\mathrm{f}}\right) P\left(\boldsymbol{x}_m^{\mathrm{f}}\right).$$

Genotype data simulation

To generate the descendent haplotype and genotype data for each dataset in each scenario, the breeding pedigree was first simulated via the following. The homoP2_F2 scenario assumes that the two inbred founders have homozygotes at all markers and are biallelic between founders, the founders are crossed and then self-pollinate, as usually conducted in the genetic analysis of self-fertilizing plants. The hetP2_F1 scenario assumes two outbred founders with independently random genotypes at any marker with the minor allele observed in at least one of the four possible haplotypes. These outbred founders are subjected to mating to produce 1000 progenies. This scenario assumes the case of genetic analyses for non-self-fertilizing organisms. The pedigree of homoP8_RIL consists of three pairing generations and five self-fertilized generations. In

the pairing stage, four pairs are selected from the eight founders with no duplication, and one descendant is created from each pair. The four descendants are then further paired to produce 100 progenies from each of the two pairs. At this step, we have two sibling families with 100 individuals in each. At the last pairing, 50 pairs are produced by selecting one individual per sibling family to produce pairs from each of which 50 progenies are generated. The resultant 2500 individuals are then self-fertilized five times so that one offspring is produced per individual to maintain the population size. This kind of pedigree is usually found in populations that have been subjected to genetic survey and breeding and known as Multiparent Advanced Generation Inter-Cross (MAGIC) population. A given number of offspring is sampled from the last generation to prepare each dataset.

The descendent haplotype data from each population were generated by introducing crossovers into the gametes that have descended from the parent(s) of each progeny at each mating step. Only one pair of 50 Mb chromosomes as a diploid genome was simulated for all datasets and the expected genetic distance per megabase pair was set to 0.04. Markers were located randomly throughout the chromosome. The occurrence of crossovers between adjacent markers in a gamete follows Poisson distribution with a mean value that is equal to the genetic distance of the given pair of adjacent markers. The genetic distances between the markers were calculated by multiplying the physical distances by 0.04×10^{-6}. If an odd number of crossovers was assigned to a marker pair, gamete switching descendent haplotypes were generated, while the assigned crossover was ignored if the donor parent of the gamete showed identical-by-descent around the markers. The offspring genotypes were obtained by replacing the descendent haplotype at each marker with the genotype that was assigned to the founder haplotype.

Read count data simulation

Generally, the total number of reads in read count data that is obtained via NGS is competitively allocated to markers of sequenced samples with biases that depend on the proportion of sequencing targets in a sequencing library. To mimic the competitiveness of reads between markers of samples, reads were allocated following the joint probability for all markers and samples as explained below.

The total number of reads per marker y_m^T is assumed to follow exponential distribution with the rate parameter λ, $\mathrm{Exp}(\lambda)$. The probability of a read at marker *m* can be obtained by the following:

$$P(m) = \frac{y_m^T}{\sum y_m^T}, y_m^T \sim \text{Exp}(\lambda),$$

where λ is set at 0.25. The read obtained at marker m can be either reference or alternative with probabilities of p^{ref} and $(1 - p^{\text{ref}})$, respectively, where p^{ref} represents the probability that the read is a reference allele. The probability p^{ref} takes different values depending on the genotype at marker m in sample i, x_{mi}. When the genotype is the homozygous reference, $p^{\text{ref}} = (1 - e^{\text{seq}})$, where e^{seq} represents a sequencing error. Similarly, $p^{\text{ref}} = 0.5$ and $p^{\text{ref}} = e^{\text{seq}}$ for heterozygous and homozygous alternatives. To incorporate allele read bias, the genotype-dependent read observation probability $P(z|w_m, x_{mi}), z \in \{\text{ref}, \text{alt}\}$ was introduced, which takes the values shown in Supplementary Table 1. The joint probability of having a read for allele z at marker m in sample i can then be calculated:

$$P(z, m, i|w_m, x_{mi}) = P(z|w_m, x_{mi})P(m)P(i), P(i) = \frac{1}{N},$$

where N is the number of samples. Mismapping error is introduced by replacing the genotypes with the probability $P\left(x'_{mi} \middle| x_{mi}, e_m^{\text{map}} = \left(e^{\text{ref}}, e^{\text{alt}}\right)\right)$, taking the values listed in Table 1. The number of total reads is calculated by multiplying the number of samples by the expected read depth and the number of markers. We then allocate those reads based on the joint probability $P(z, m, i|w_m, x'_{mi})$, which is not conditional on the true genotype x_{mi}. The read count data for the founders and the offspring are simulated separately with given expected read depths for each dataset.

$P(m)$ was obtained from the real data by calculating the total number of reads per marker y_m^T by summing up the read counts of each marker. The values w_m were obtained by calculating $y_m^{\text{ref}}/(y_m^{\text{ref}} + y_m^{\text{alt}})$, where y_m^{ref} and y_m^{alt} denote the total numbers of reference and alternative reads per marker observed in the real data, respectively. Mismapping rates were randomly assigned to markers by sampling the probabilities from an exponential distribution with the mean at 0.05, since the mismapping pattern in the real data is unknown. The sequencing error rate e^{seq} was set to 0.0025.

Input parameter settings

GBScleanR includes some arguments that can be used to tweak this tool: the expected genetic distance per megabase pair E^d, sequencing error rate e^{seq}, the threshold for the probability of calling estimated genotype P_{call}, and the number of iterations required for genotype estimation. To decide the default values for these arguments, we tested the

algorithm using the simulation dataset with 100 individuals and a 3x offspring read depth in the “nonzero” dataset of the homoP2_F2 scenario under various settings. As shown in Supplementary Fig. 2, the tests indicated that P_{call} has a relatively large impact on the genotype estimation accuracy, while the differences associated with the number of iterations (if more than one), E^d, and e^{seq} were negligible. The values of $E^d = 0.04$ and $e^{seq} = 0.0025$ that were used in the data simulation seem to be good choices when set for genotype estimation. Increasing P_{call} from 0.8 to 0.95 improved the correct call rate in an almost proportional manner, while the miscall rate worsened at the same time. Therefore, we set P_{call} at 0.9 for the algorithm evaluation as used in the default setting for magicImpute. In the case of iteration number, we found that a larger number leads to better correct call and miscall rates, which reached a plateau at four or more iterations. Thus, we decided to set the number of iterations to four. To evaluate GBScleanR, we tested the algorithm with and without the iterative parameters optimization (IPO) w_m and e_m^{map}.

For fair comparison among the three tools, similar input parameters were used if LB-Impute and magicImpute have corresponding parameters to those given in GBScleanR. In the case of LB-impute, the "impute" option was set to the "-method" argument and the "-offspringimpute" flag was used to run it in the offspring impute mode. A vector of strings stating the names of the founder samples was specified as "-parents". “-recombdist” and “-readerr” were set at 12500000 and 0.0025, which correspond to E_d and e^{seq}. While the parameter related to read mismapping is estimated by IPO and not tunable in GBScleanR, LB-Impute requires setting the parameter “-genotypeerr” that is related to e^{map} of GBScleanR. We set 0.005 to “-genotypeerr” as magicImpute also requires similar parameters and has 0.005 as default. The arguments in magicImpute include the mandatory arguments "model" and "popdesign" in addition to the genotype data in the "magicsnp" format. The simulated data and real data were converted to the magicsnp format. Marker positions in centimorgan, which are required for magicImpute, were calculated by multiplying the physical positions of the markers by the expected genetic distance per megabase of 0.04, which was used to generate the simulation data. The "jointModel" option was set to the "model" argument for all datasets. The "popdesign" argument that affects the state transition rates was specified based on the pedigree in each scenario as follows. The “popdesign” was set to ("Pairing", "Selfing") for the scenario homoP2_F2, ("Pairing") for the scenario hetP2_F1, and ("Pairing", "Pairing", "Pairing", "Selfing", "Selfing", "Selfing", "Selfing", "Selfing") for homoP8_RIL. Additionally, magicImpute has “minPhredQualScore”, “imputingThreshold”, and “detectingThreshold” arguments. The

former corresponds to e^{seq} and was set at 26.0206 that is equal to $e^{seq} = 0.0025$. The latter two specify the parameter similar to P_{call} of GBScleanR and both of them were set at 0.9. Both "founderAllelicError" and "offspringAllelicError" that corresponds to e^{map} of GBScleanR and "-genotypeerr" of LB-Impute were set to 0.005. The estimated genotype data generated by magicImpute includes incomplete genotype estimations represented by 1N, N1, 2N, and N2, where N points to an unknown allele and the numbers 1 and 2 indicate reference and alternative alleles, respectively. Although these incomplete estimations provide partial information about the genotype at the markers, we treat them as completely missing because downstream genetic analyses generally cannot handle such incomplete information.

Supplementary Results

Estimation of founder genotype and phased genotype

We compared the founder genotype estimation accuracy of the algorithms. As expected from the estimation accuracy for offspring, GBScleanR outperformed magicImpute (Supplementary Fig. 6 and Supplementary Data 3). While both algorithms showed similar and high correct call rates in the "nonezero" datasets of homoP2_F2 and homoP8_RIL, magicImpute resulted in less accurate founder genotype estimations compared with GBScleanR for the "allowzero" datasets (Supplementary Fig. 6a-c and g-i). Unlike the offspring genotype estimation, IPO did not affect the founder genotype estimations in the homoP2_F2 and homoP8_RIL datasets (Supplementary Fig. 6a-c and g-i). Improvement in the estimation accuracy by IPO was observed only in the hetP2_F1 datasets with a relatively large number of offspring and offspring reads (Supplementary Fig. 6d-f). This might be because heterozygotes in the founder genotypes were only assumed in the hetP2_F1 datasets but not in the others; the homozygous genotypes of the inbred founders were not affected by allele read biases.

In addition to the genotype information of offspring and founders, both GBScleanR and magicImpute provide phasing information for the offspring genotypes. Phased genotype, which is also referred to as haplotype, is the sequence of alleles on each of a chromosome pair. Correct call rates were evaluated by scoring the proportion of estimated haplotypes matching the simulated haplotypes. As expected from the superiority for both founder and offspring genotype estimations, GBScleanR also outperformed magicImpute in the haplotype estimations (Supplementary Fig. 7 and Supplementary Data 4).

Supplementary Table 1 Genotype-dependent read observation probabilities

		Observable allele read z	
		Ref	Alt
Simulated genotype x_{mi}	0	$(1-e^{\mathrm{seq}})w_m$	$e^{\mathrm{seq}}w_m$
	1	$0.5w_m$	$0.5(1-w_m)$
	2	$e^{\mathrm{seq}}(1-w_m)$	$(1-e^{\mathrm{seq}})(1-w_m)$

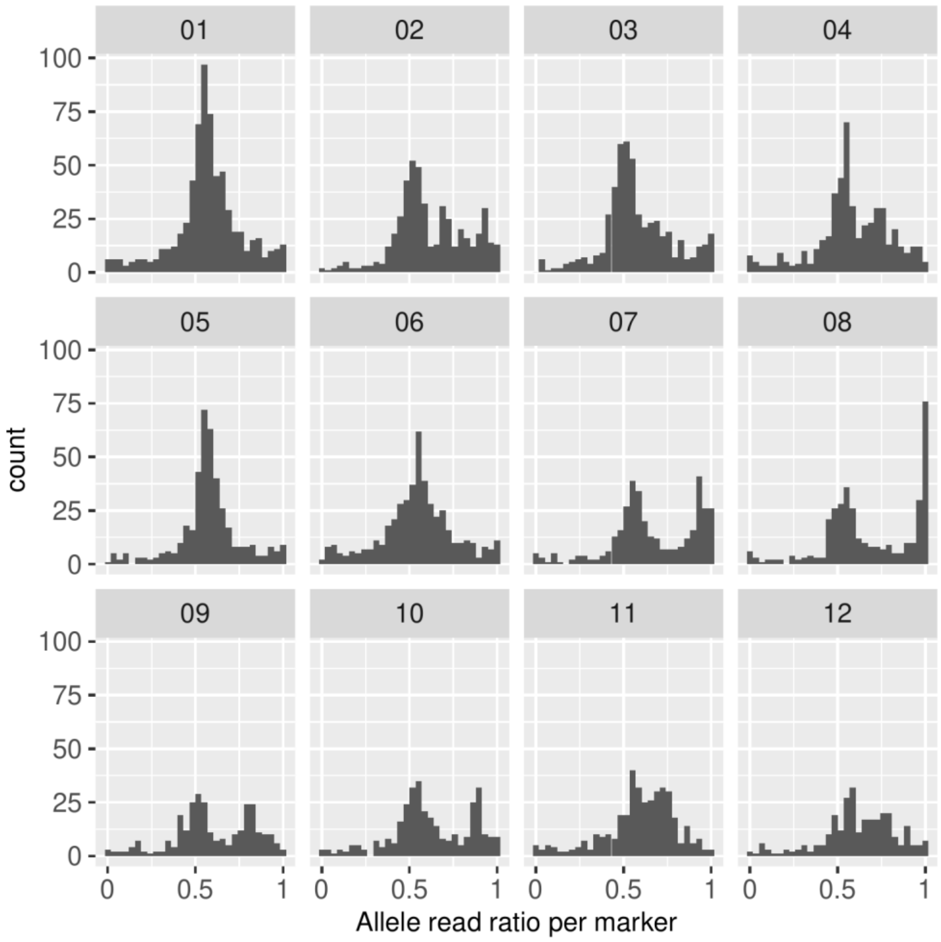

Supplementary Fig. 1. Allele read ratio per marker observed in the real data

The histograms of the allele read ratios calculated for each marker are plotted separately for chromosomes 1-12. The allele read ratio greater than 0.5 indicates a preferable observation of reference allele reads at the marker. These histograms indicate that many markers tend to have reference reads. The majority of the markers counted in the right-most bin of each histogram were associated with high missing rates, which may lead to apparent allele read biases. The markers in the real data seem to prefer reference allele reads in all chromosomes.

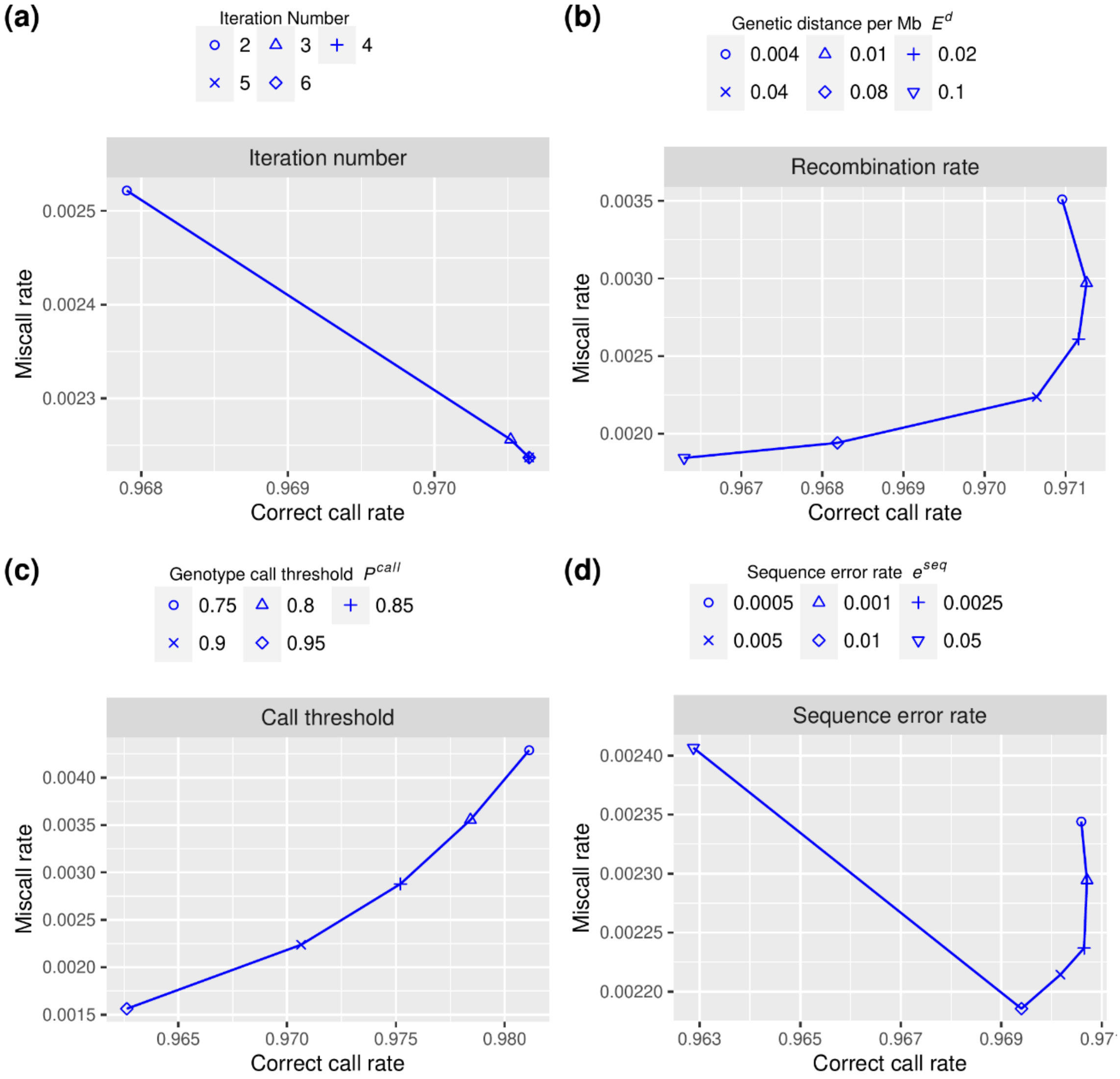

Supplementary Fig. 2. Effects of parameter setting on genotype estimation accuracy. Correct call rates and miscall rates at various parameter settings are plotted for the iteration number (a), the expected genetic distance per megabase pair E^d (b), the genotype call threshold P_{call} (c), and the sequencing error rate e^{seq} (d). Different symbols indicate different settings for each parameter.

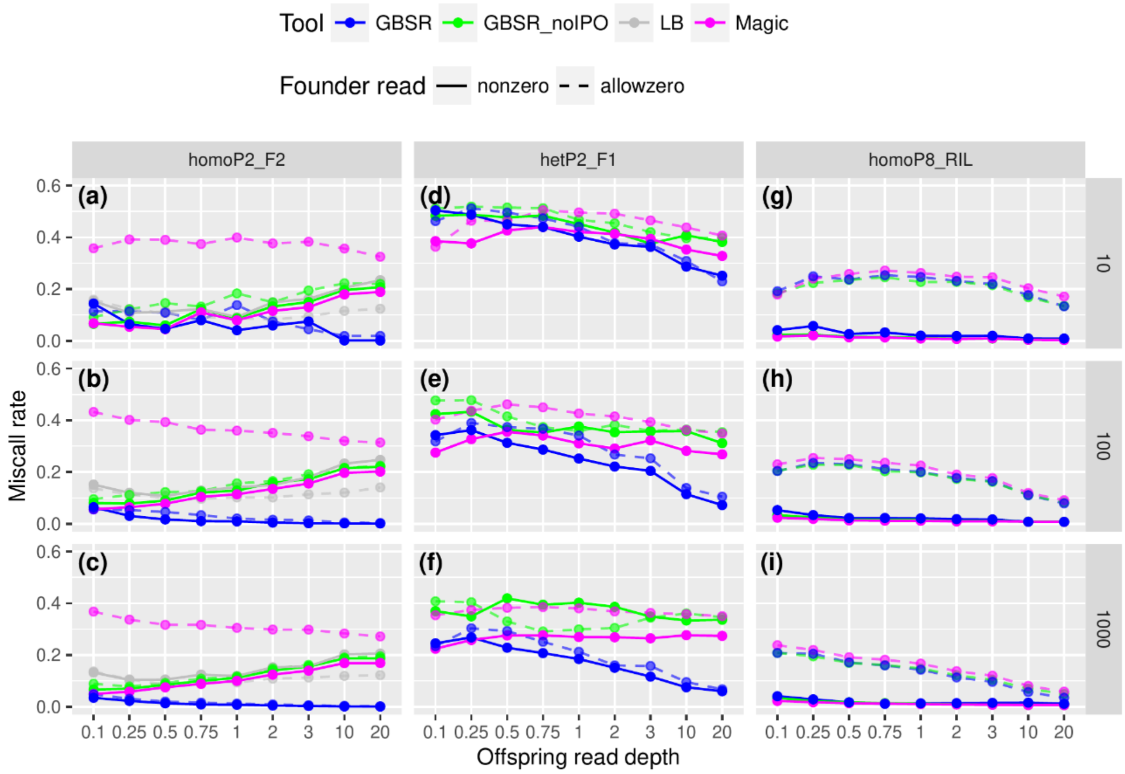

Supplementary Fig. 3. Miscall rates in offspring genotype estimation for the simulation data.

The plots show miscall rates for the datasets with given offspring read depths (x-axis) in the homoP2_F2 (a-c), hetP2_F1 (d-f), and homoP8_RIL (g-i) scenarios. The number of samples in each simulated dataset is indicated in the strips on the right. Solid lines and dashed lines represent the results for the datasets with (allowzero) or without (nonzero) missing in founder read counts. “GBSR” and “GBSR_noIPO” represent GBScleanR with and without IPO, respectively, while “LB” and “Magic” indicate LB-Impute and magicImpute, respectively.

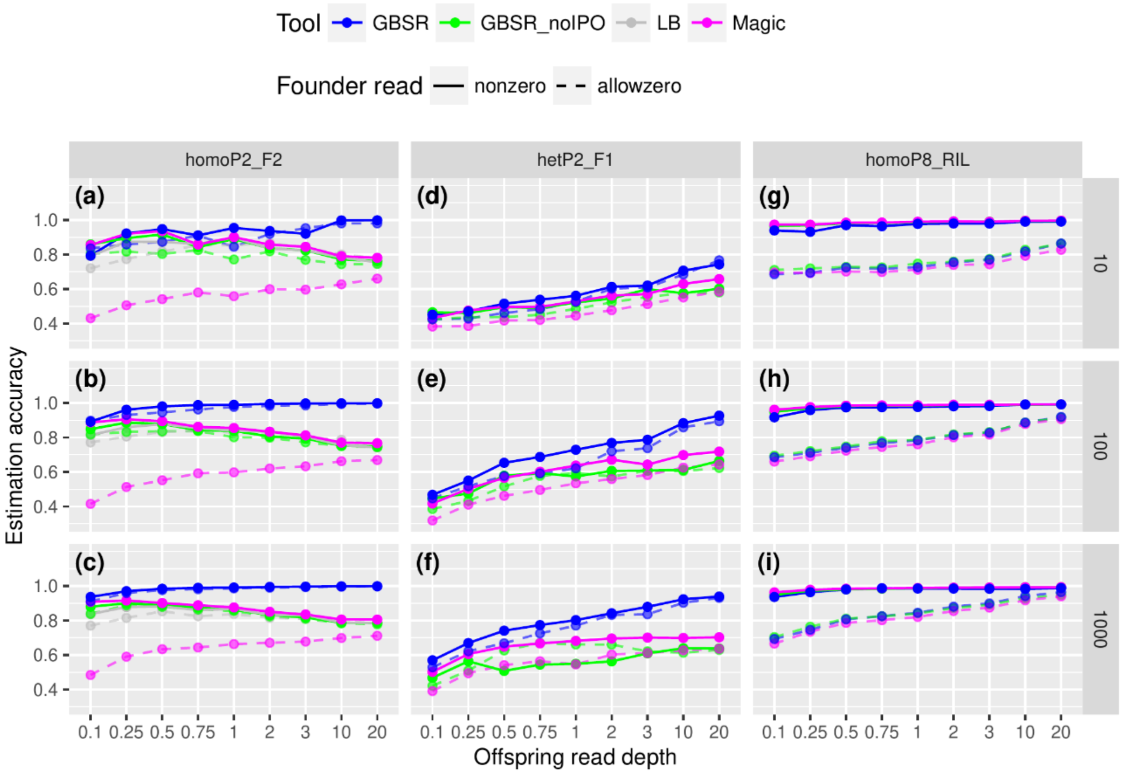

Supplementary Fig. 4. Accuracy in offspring genotype estimation for the simulation data.

The plots show estimation accuracy (correct call rate / non-missing call rate) for the datasets with given offspring read depths (x-axis) in the homoP2_F2 (a-c), hetP2_F1 (d-f), and homoP8_RIL (g-i) scenarios. The number of samples in each simulated dataset is indicated in the strips on the right. Solid lines and dashed lines represent the results for the datasets with (allowzero) or without (nonzero) missing in founder read counts. "GBSR" and "GBSR_noIPO" represent GBScleanR with and without IPO, respectively, while "LB" and "Magic" indicate LB-Impute and magicImpute, respectively.

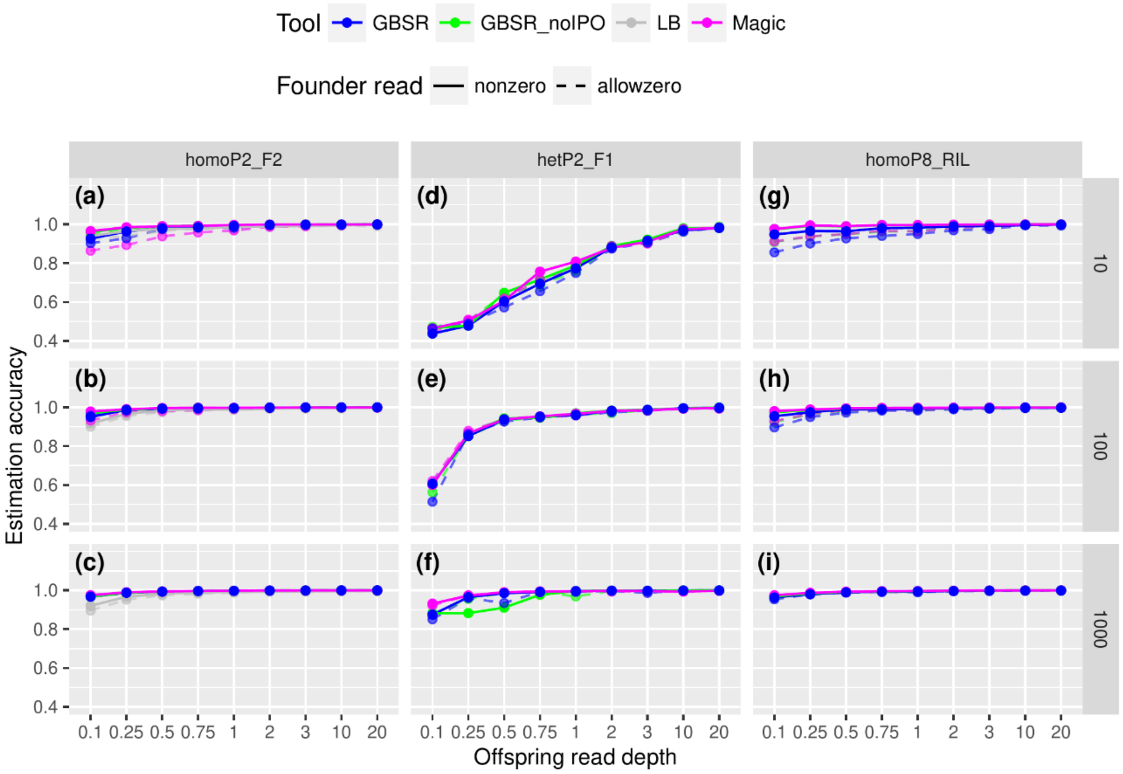

Supplementary Fig. 5. Accuracy in offspring genotype estimation for the simulation data without bias.

The plots show estimation accuracy (correct call rate / non-missing call rate) for the datasets that were simulated without assuming allele read bias with given offspring read depths (x-axis) in the homoP2_F2 (a-c), hetP2_F1 (d-f), and homoP8_RIL (g-i) scenarios. The number of samples in each simulated dataset is indicated in the strips on the right. Solid lines and dashed lines represent the results for the datasets with (allowzero) or without (nonzero) missing in founder read counts. “GBSR” and “GBSR_noIPO” represent GBScleanR with and without IPO, respectively, while “LB” and “Magic” indicate LB-Impute and magicImpute, respectively.

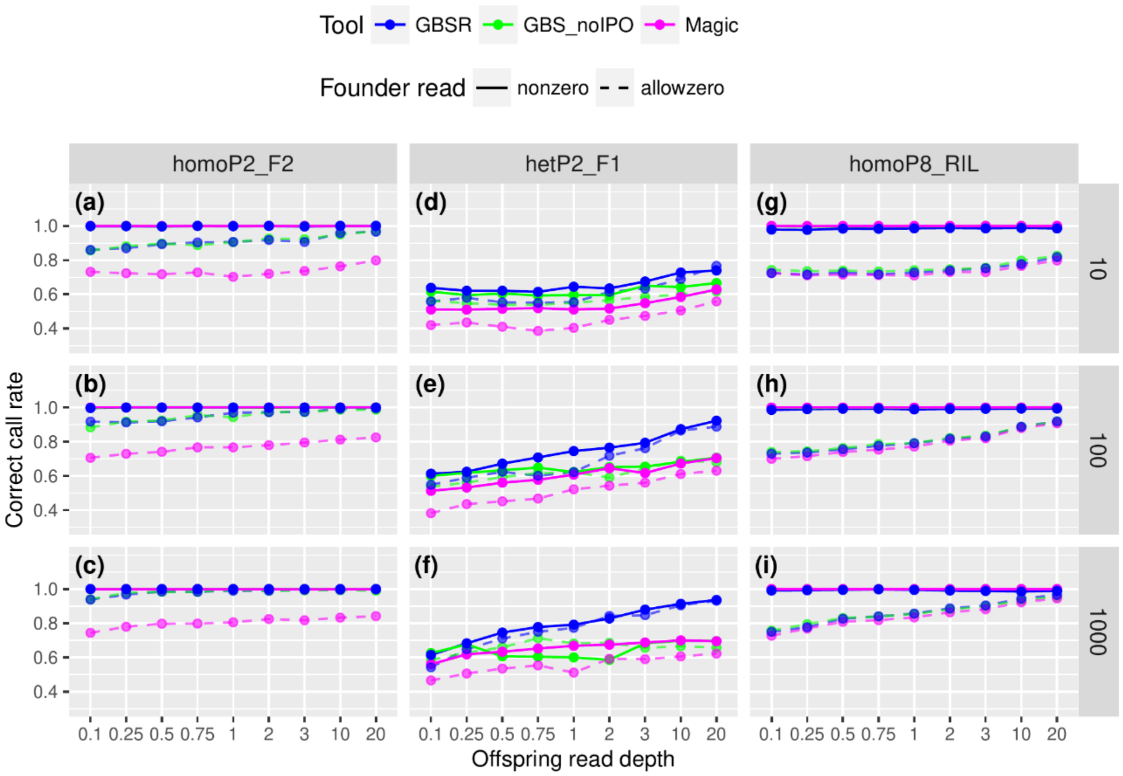

Supplementary Fig. 6. Correct call rates in founder genotype estimation for the simulation data.

The plots show correct call rates for the datasets with given offspring read depths (x-axis) in the homoP2_F2 (a-c), hetP2_F1 (d-f), and homoP8_RIL (g-i) scenarios. The number of samples in each simulated dataset is indicated in the strips on the right. Solid lines and dashed lines represent the results for the datasets with (allowzero) or without (nonzero) missing in founder read counts. “GBSR” and “GBSR_noIPO” represent GBScleanR with and without IPO, respectively, while “LB” and “Magic” indicate LB-Impute and magicImpute, respectively.

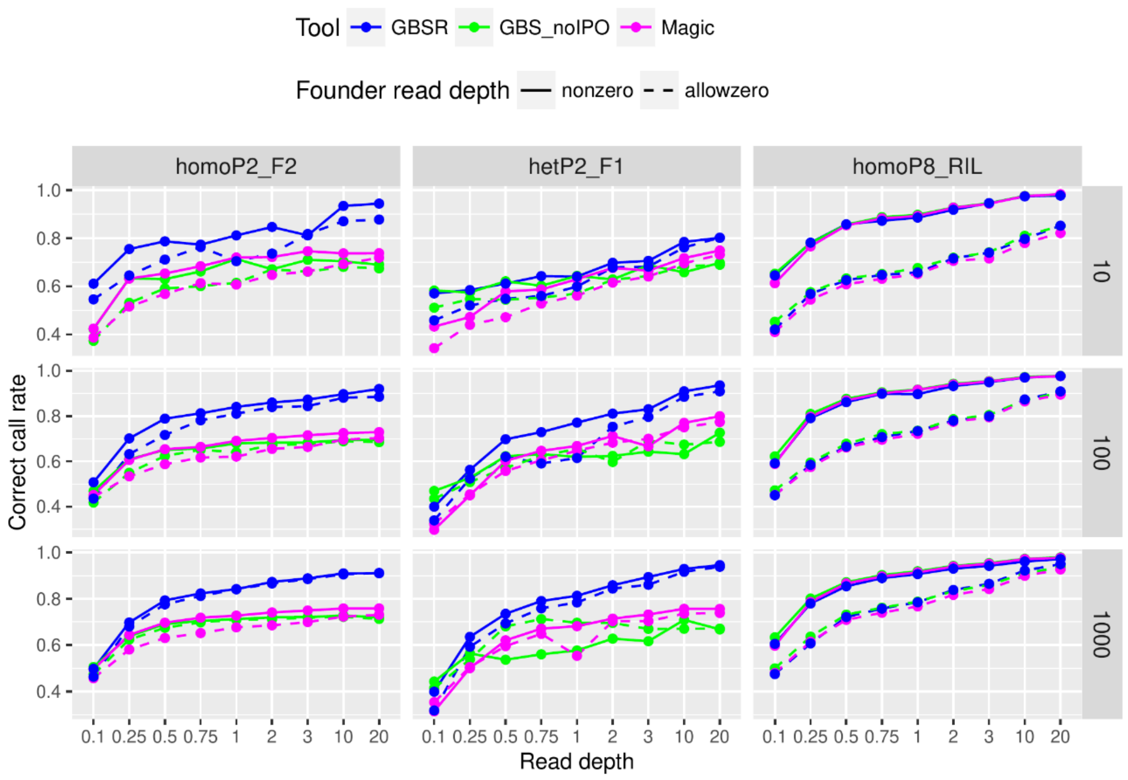

Supplementary Fig. 7. Correct call rates in offspring haplotype estimation for the simulation data.

The plots show correct call rates of haplotypes for the datasets with given offspring read depths (x-axis) in the homoP2_F2 (a-c), hetP2_F1 (d-f), and homoP8_RIL (g-i) scenarios. The number of samples in each simulated dataset is indicated in the strips on the right. Solid lines and dashed lines represent the results for the datasets with ("allowzero") or without ("nonzero") missing in founder read counts. "GBSR" and "GBSR_noIPO" represent GBScleanR with and without IPO, respectively, while "Magic" indicates magicImpute.

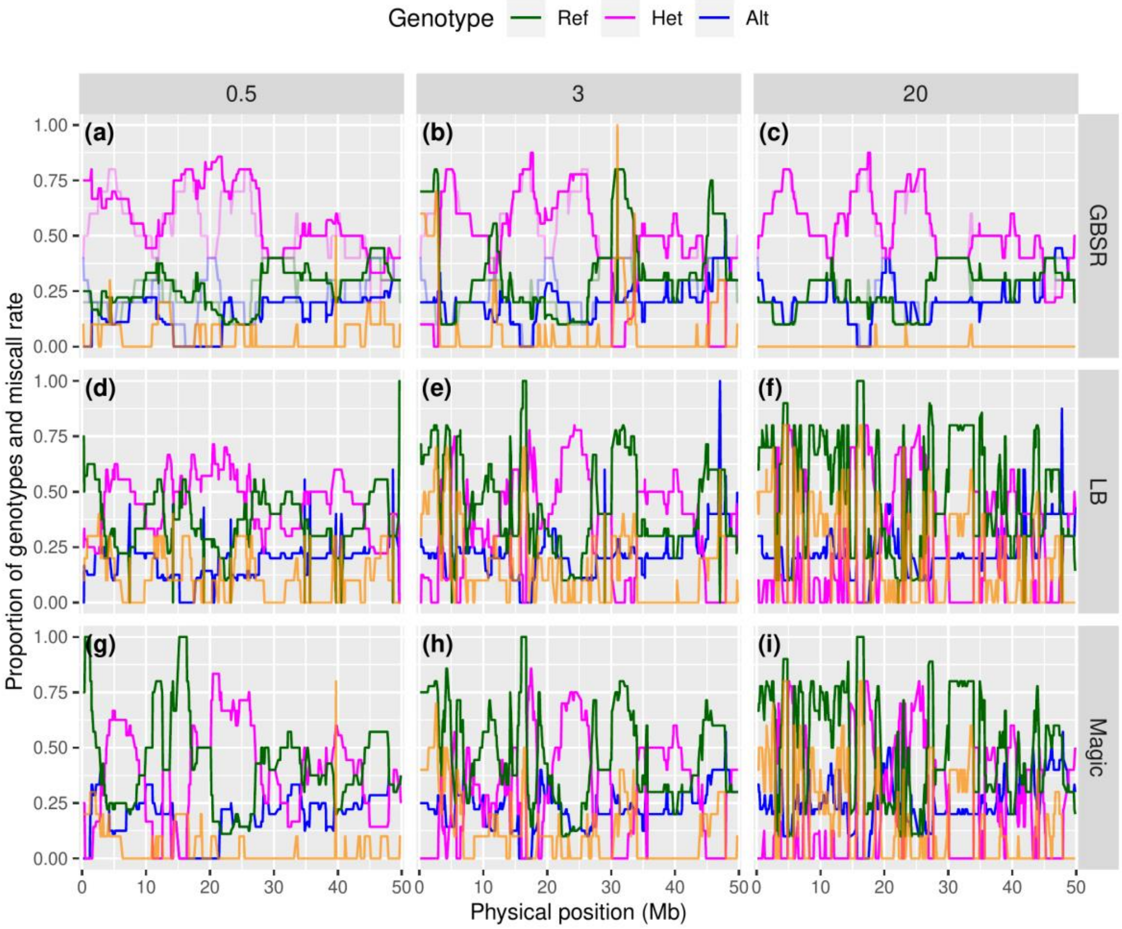

Supplementary Fig. 8. Genotype ratios at markers ordered along a simulated chromosome.

The plots show genotype ratios calculated from the estimated genotypes for the homoP2_F2 dataset with 10 offspring and 620 markers with offspring read depths at 0.5×, 3×, and 20× without allowance of no reads in founders. "GBSR" (a-c), "LB" (d-f), and "Magic" (g-i) indicate genotypes estimated by GBScleanR, LB-Impute, and magicImpute, respectively. Proportions of reference homozygous, heterozygous, and alternative homozygous genotypes are represented by green, magenta, and blue lines, respectively. Orange lines indicate the miscall rates at markers. True genotype ratios are indicated by transparent lines in the panels of GBScleanR (a-c).

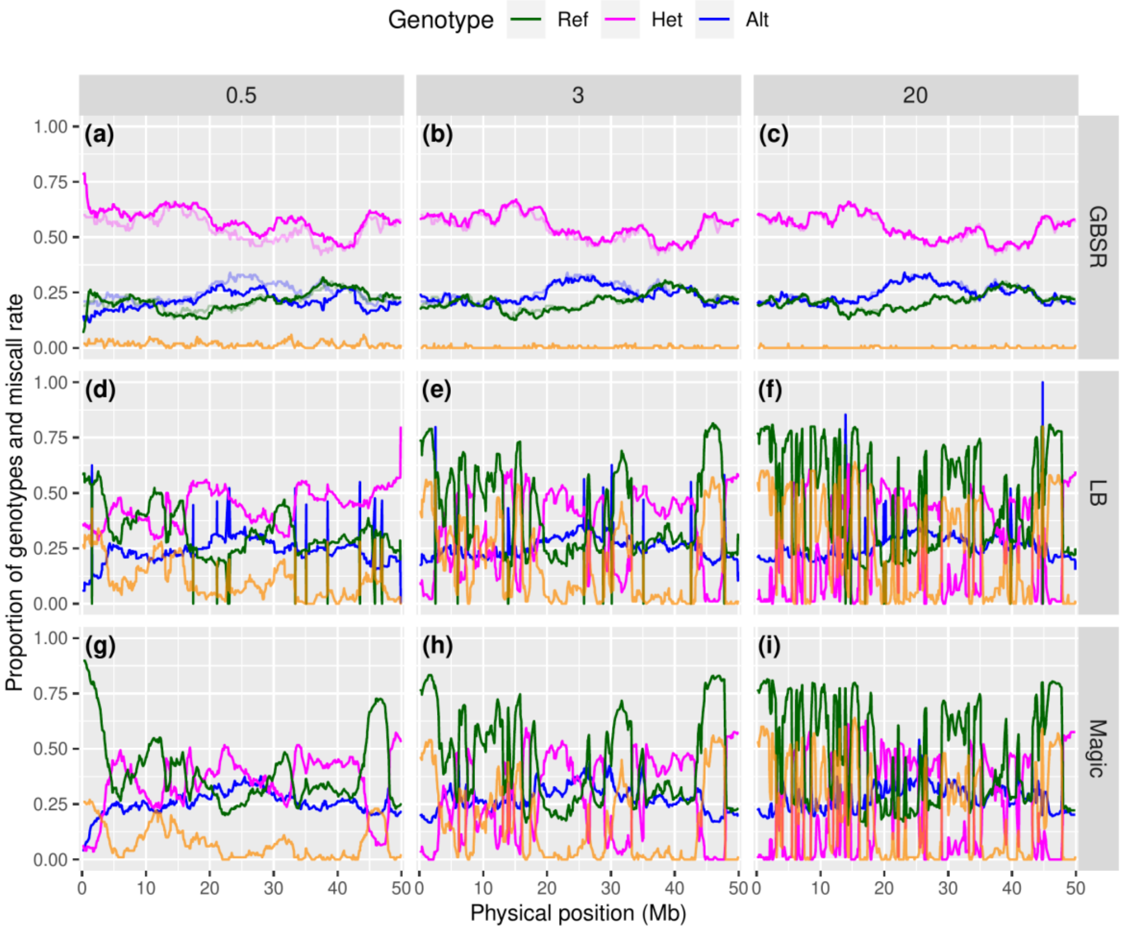

Supplementary Fig. 9. Genotype ratios at markers ordered along a simulated chromosome.

The plots show genotype ratios calculated from the estimated genotypes for the homoP2_F2 dataset with 100 offspring and 620 markers with offspring read depths at 0.5×, 3×, and 20× without allowance of no reads in founders. “GBSR” (a-c), “LB” (d-f), and “Magic” (g-i) indicate genotypes estimated by GBScleanR, LB-Impute, and magicImpute, respectively. Proportions of reference homozygous, heterozygous, and alternative homozygous genotypes are represented by green, magenta, and blue lines, respectively. Orange lines indicate the miscall rates at markers. True genotype ratios are indicated by transparent lines in the panels of GBScleanR (a-c).

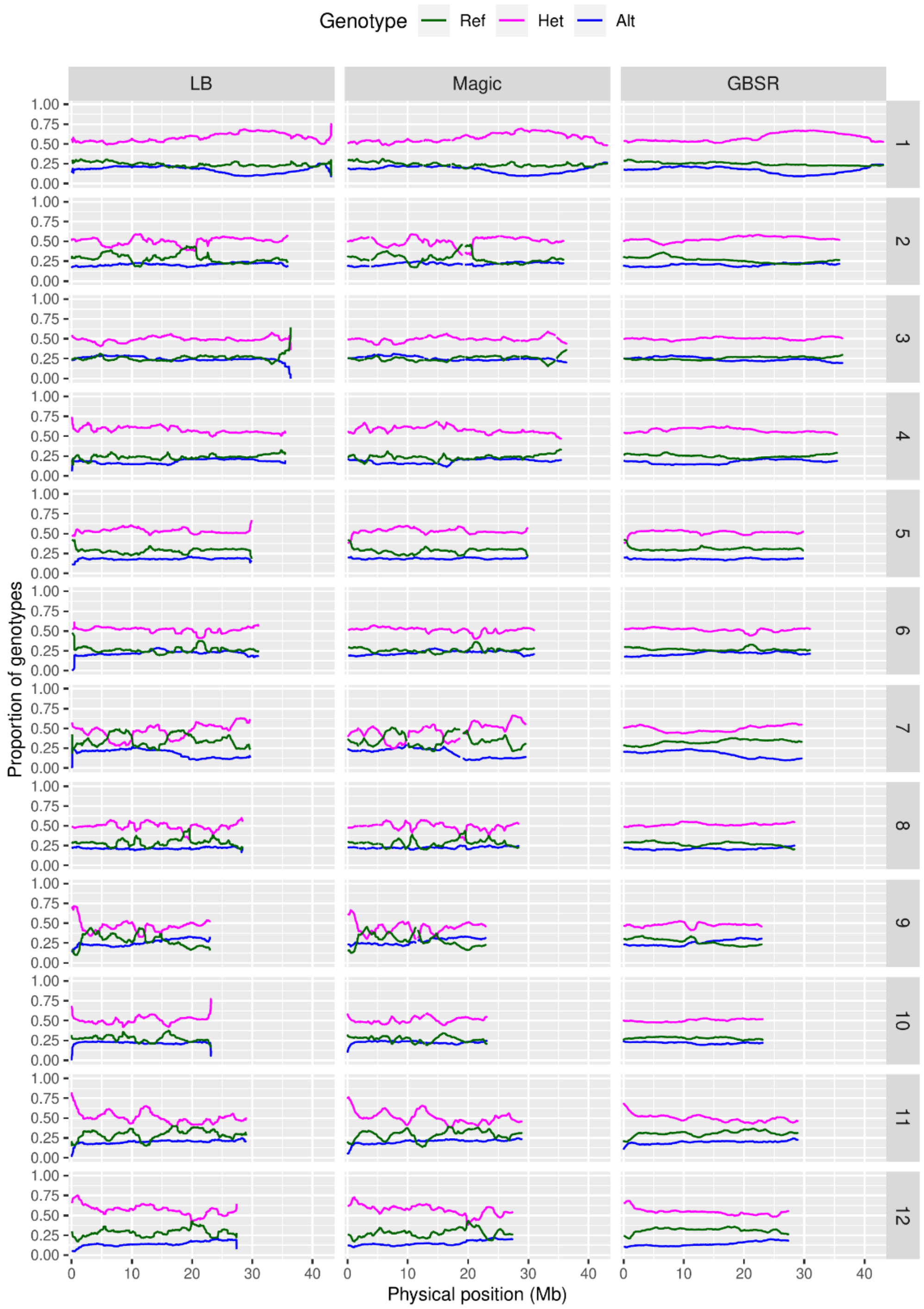

Supplementary Fig. 10. Genotype ratios of estimated offspring genotypes for all chromosomes in the real data.

Genotype ratios for 12 chromosomes are shown in the line plots. “GBSR”, “LB”, and “Magic” indicate GBScleanR, LB-Impute, and magicImpute, respectively. Proportions of reference homozygous, heterozygous, and alternative homozygous genotypes are represented by green, magenta, and blue lines, respectively.

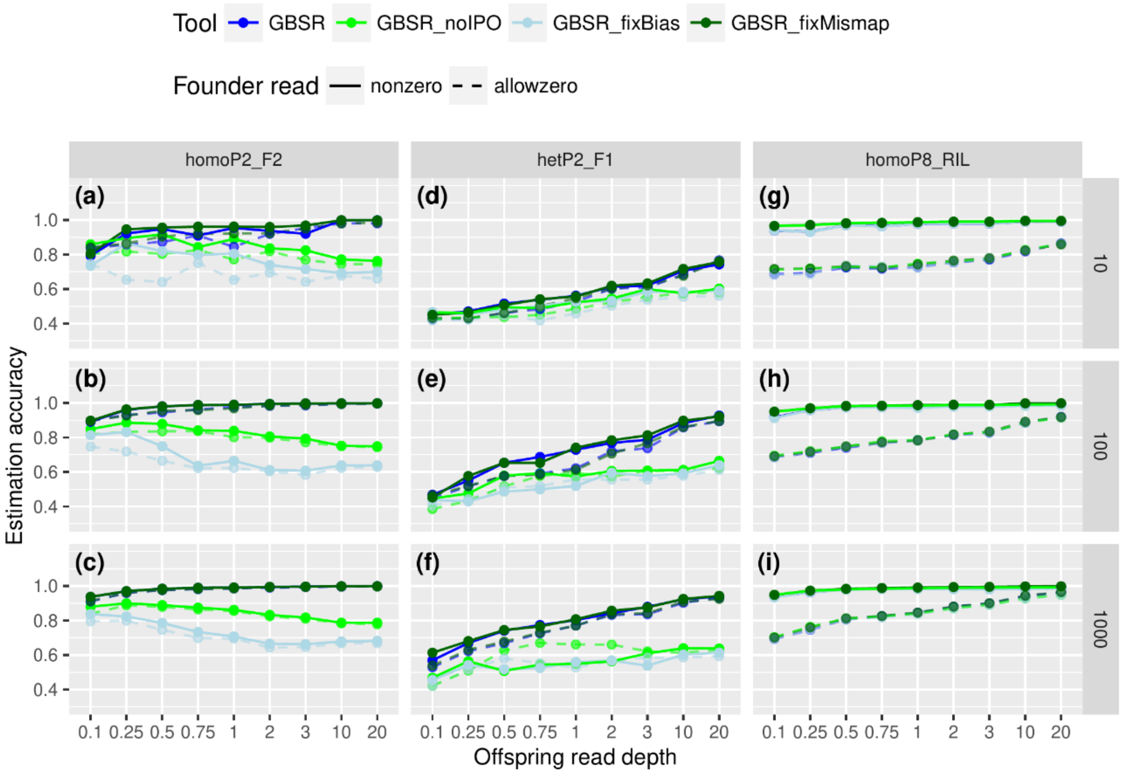

Supplementary Fig. 11. Accuracy in offspring genotype estimation for the simulation data using GBScleanR with different IPO settings.

The plots show the estimation accuracy for the datasets with given read depths for offspring (x-axis) in the homoP2_F2 (a-c), hetP2_F1 (d-f), and homoP8_RIL (g-i) scenarios. The rows of the panels indicate the differences in the number of samples in the simulated data, as shown in the strips on the right. Solid lines and dashed lines represent the results for the datasets with (allowzero) or without (nonzero) missing founder reads. "GBSR" and "GBSR_noIPO" represent GBScleanR with and without IPO. "GBSR_fixBias" indicates GBScleanR with a fixed allele read bias and the iterative optimization only for mismapping rates, whereas "GBSR_fixMismap" means GBScleanR with a fixed mismapping rate and the iterative optimization only for allele read biases.

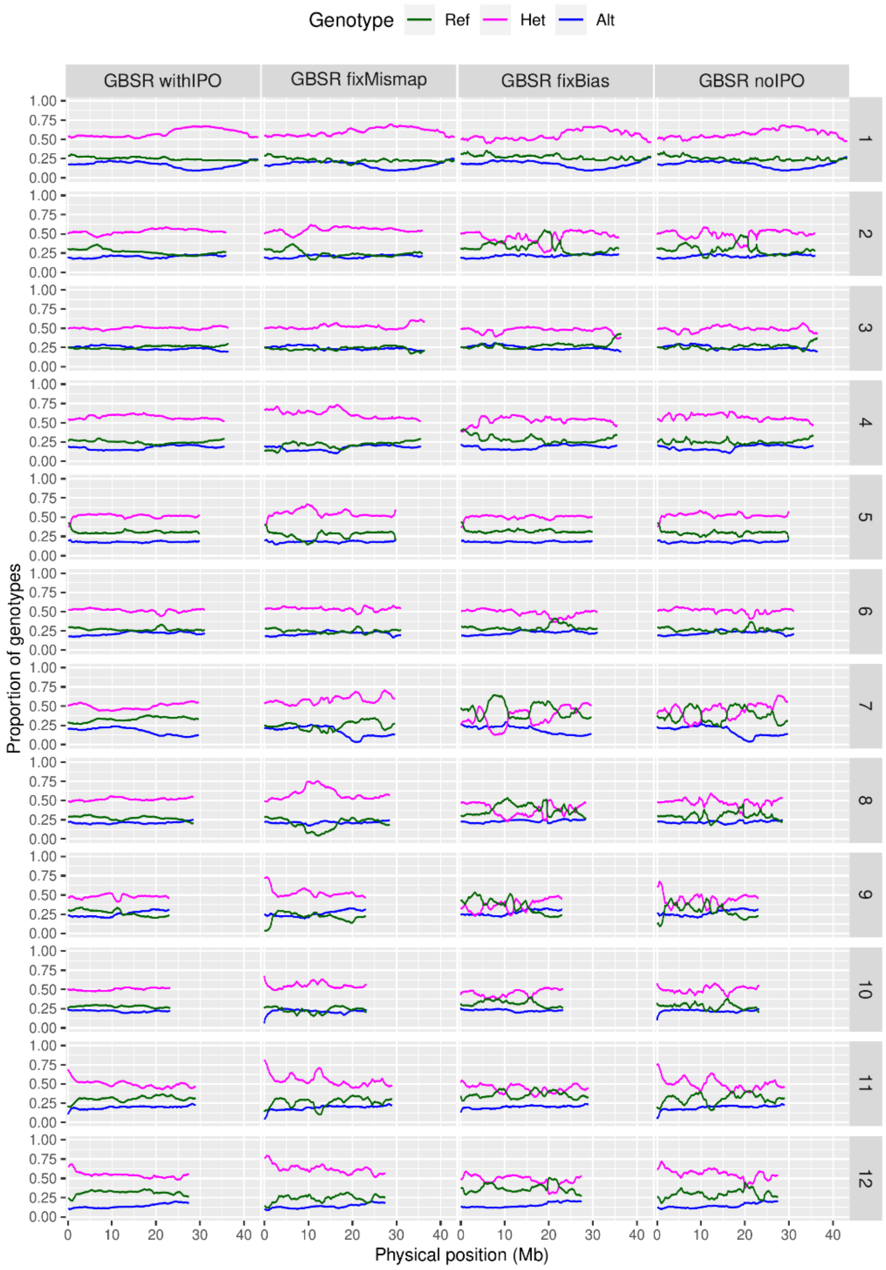

Supplementary Fig. 12. Genotype ratios of estimated offspring genotypes in the real data using GBScleanR with different IPO settings.

Genotype ratios for 12 chromosomes are shown in the line plots. Proportions of reference homozygous, heterozygous, and alternative homozygous genotypes are represented by green, magenta, and blue lines, respectively. “GBSR withIPO” and “GBSR noIPO” represent GBScleanR with and without IPO. “GBSR fixMismap” means GBScleanR with a fixed mismapping rate and the iterative optimization only for allele read biases, whereas “GBSR fixBias” indicates GBScleanR with a fixed allele read bias and the iterative optimization only for mismapping rates.

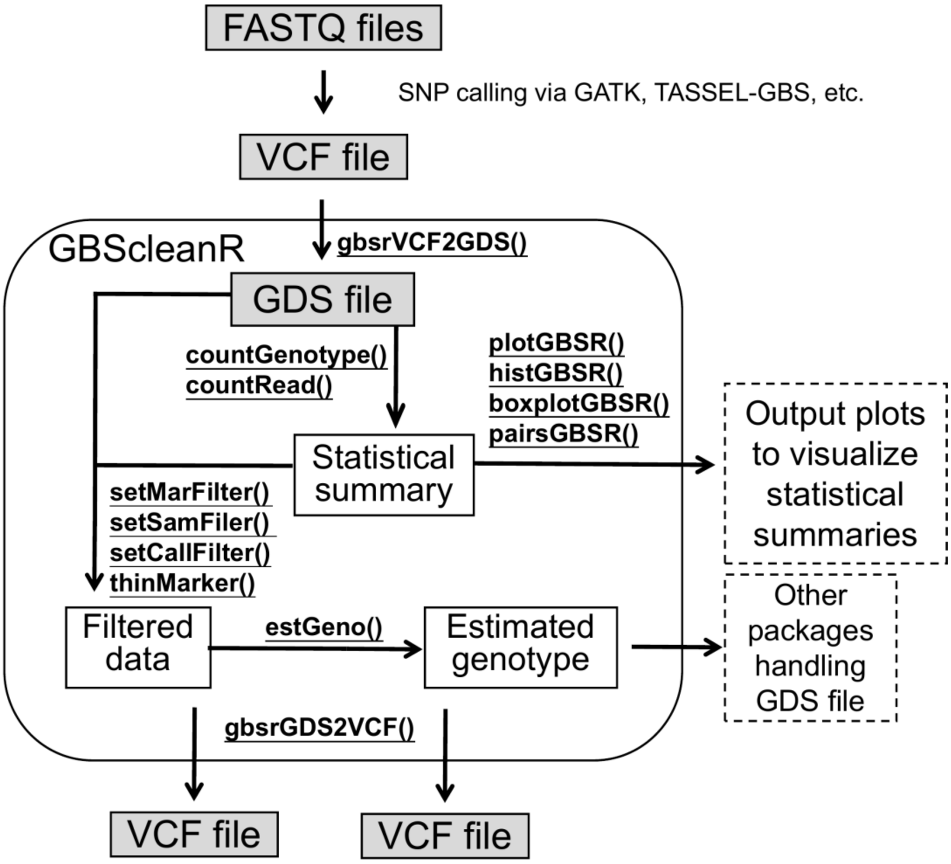

Supplementary Fig. 13. Schematic image of the workflow with GBScleanR.
Gray boxes represent data files, while white boxes are data created by GBScleanR in the R environment. Underlined texts indicate the function names of GBScleanR. GBScleanR takes a VCF file generated via SNP callers such as GATK and TASSEL-GBS as inputs. An input VCF file first is converted to a GDS file to handle large genotype data on the R environment with less RAM usage. Statistical summaries of genotype and read counts can be obtained via countGenotype() and countRead(). The summary data can be then visualized using several plotting functions. Users can also filter out samples and markers based on the statistical summaries using setMarFilter() and setSamFilter(). setCallFilter() allows users to filter out single genotype calls at markers based on read counts, while thinMarker() retains one of the markers within a specified interval to reduce the number of markers with redundant information. estGeno() is the core function of GBScleanR that estimates genotypes based on given read counts. The filtered genotype data and the estimated genotype data can be output as a VCF file by gbsrGDS2VCF().
